# Dynamic structure of T4 gene 32 protein filaments facilitates rapid noncooperative protein dissociation

**DOI:** 10.1093/nar/gkad595

**Published:** 2023-07-14

**Authors:** Ben A Cashen, Michael Morse, Ioulia Rouzina, Richard L Karpel, Mark C Williams

**Affiliations:** Department of Physics, Northeastern University, Boston, MA 02115, USA; Department of Physics, Northeastern University, Boston, MA 02115, USA; Department of Chemistry and Biochemistry, Center for Retroviral Research and Center for RNA Biology, Ohio State University, Columbus, OH 43210, USA; Department of Chemistry and Biochemistry, University of Maryland Baltimore County, Baltimore, MD 21250, USA; Department of Physics, Northeastern University, Boston, MA 02115, USA

## Abstract

Bacteriophage T4 gene 32 protein (gp32) is a model single-stranded DNA (ssDNA) binding protein, essential for DNA replication. gp32 forms cooperative filaments on ssDNA through interprotein interactions between its core and N-terminus. However, detailed understanding of gp32 filament structure and organization remains incomplete, particularly for longer, biologically-relevant DNA lengths. Moreover, it is unclear how these tightly-bound filaments dissociate from ssDNA during complementary strand synthesis. We use optical tweezers and atomic force microscopy to probe the structure and binding dynamics of gp32 on long (∼8 knt) ssDNA substrates. We find that cooperative binding of gp32 rigidifies ssDNA while also reducing its contour length, consistent with the ssDNA helically winding around the gp32 filament. While measured rates of gp32 binding and dissociation indicate nM binding affinity, at ∼1000-fold higher protein concentrations gp32 continues to bind into and restructure the gp32–ssDNA filament, leading to an increase in its helical pitch and elongation of the substrate. Furthermore, the oversaturated gp32–ssDNA filament becomes progressively unwound and unstable as observed by the appearance of a rapid, noncooperative protein dissociation phase not seen at lower complex saturation, suggesting a possible mechanism for prompt removal of gp32 from the overcrowded ssDNA in front of the polymerase during replication.

## Graphical Abstract

**Graphical Abstract:**
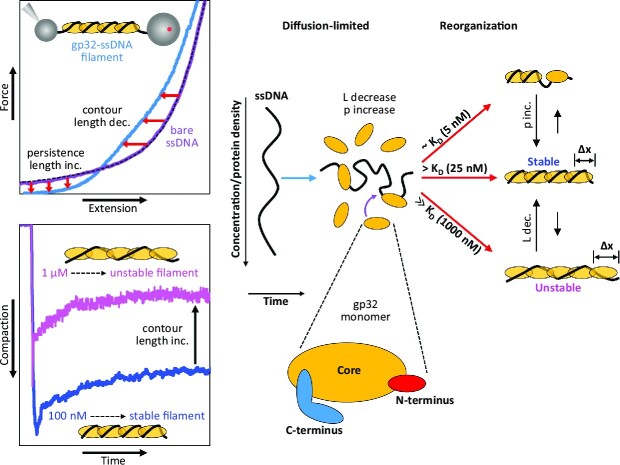

## INTRODUCTION

T4 bacteriophage is a virulent phage species that infects *Escherichia coli*. Its DNA replication system closely resembles those of higher organisms and can therefore serve as a useful model for understanding the central features of these highly complex systems (1–4). The T4-coded replication complex consists of the three major subassemblies characteristic of all higher organisms: the DNA polymerase, the helicase-primase containing primosome, and the processivity clamp-clamp loader (1,4). The T4 single-stranded DNA (ssDNA) binding protein (SSB), gene 32 protein (gp32), is integral in the regulation and proper functioning of these components and therefore plays an essential role in T4 replication and repair (5). gp32 binds regions of ssDNA transiently produced during replication with high affinity and cooperativity, forming stable protein filaments (clusters) which serve to protect the leading and lagging strands from degradation by nucleases, as well as alter the ssDNA conformation to help optimize its interactions with T4 DNA polymerases and other replication proteins. Because it binds preferentially to ssDNA, gp32 is able to destabilize secondary structures that would otherwise inhibit polymerase functionality. Additionally, once the ssDNA is fully complexed, gp32 autogenously regulates its concentration within the host cell by binding to, and thereby suppressing translational synthesis from, its parent mRNA transcript (6–8).

gp32 is a 33.5 kDa monomer comprising three distinct domains: a central core that contains the ssDNA binding site, a positively-charged N-terminal domain (NTD) responsible for homotypic protein interactions, and a negatively-charged C-terminal domain (CTD), which has been implicated in heterotypic protein interactions (9). The core domain (residues 22–253) binds ssDNA in a positively-charged cleft created by an oligonucleotide-oligosaccharide binding fold (OB-fold), a structural motif shared by other SSBs. This cleft confers gp32 with largely sequence-independent binding and the ability to effectively discriminate against duplexed double-stranded DNA (dsDNA) (10,11). gp32 proteins bind ssDNA in a head-to-rear orientation required for cooperative interactions and the formation of stable protein filaments. The N-terminus (residues 1–21) is essential for this cooperative binding, which arises from protein-protein contacts between the NTD of a nucleic acid-bound monomer and the core domain of an adjacently bound protein (12,13). Removal of the NTD through limited tryptic digestion results in a noncooperative truncate, *II, which binds ssDNA with reduced overall affinity. The acidic CTD (residues 254–301), on the other hand, modulates interactions with other constituents of the T4 replication, repair, and recombination machinery (5,14,15).

Crystallization of gp32-DNA complexes has proven difficult (10), and thus the structural details of these complexes have yet to be entirely determined. An x-ray structure of the gp32 core (ssDNA binding) domain complexed to a short dT_6_ ssDNA lattice showed only weak electron density for the DNA within the protein's binding cleft, making it impossible to resolve the entire ssDNA oligo (10). The authors, however, were able to model four nucleotides of the dT_6_ chain into the gp32 core domain, and the resulting structure suggested that at least two nucleotides were tightly bound within the cleft. This finding was recapitulated in recent work by Jose et al., which showed that 2–3 nt were directly involved in a tight binding interaction between the ssDNA and the gp32 core domain (16). These and other studies have made considerable progress in describing the interactions of gp32 with short ssDNA substrates, which has helped extend our understanding of the dynamics and structural details of these complexes. Using 2-AP probes within poly-dT ssDNA lattices, Camel et al. mapped the local interactions between the DNA and the gp32 binding cleft at single nucleotide resolution (17). In combination with what is known from crystallographic studies, these results formed a cohesive model of the molecular interactions between gp32 and ssDNA. However, such binding studies carried out with short ssDNA substrates are limited to the interactions of DNA with either single noncontiguous monomers or small clusters thereof (*i.e*. 2–3 contiguously-bound proteins), limiting protein cooperativity. Longer, biologically-relevant DNA lengths are critical for a complete understanding of gp32 filament structure and organizational dynamics. In this regard, single molecule DNA stretching methods have allowed for significant advancement in our understanding of gp32 behavior. Binding measurements on overstretched λ-DNA demonstrated how conformational changes of the C-terminal arm (between an open and closed state) regulate salt-independent binding of gp32 to ssDNA (18–21). These studies also helped explain the origin of the ‘kinetic block’ to dsDNA melting by full-length gp32 that was observed in thermal melting experiments. However, DNA stretching experiments relying on force-melting of dsDNA require the DNA to be held at artificially high forces, limiting measurements of structural dynamics under physiological conditions. Here, we utilize a long, fully ssDNA substrate, which can be observed over a wide range of tensions, to characterize the structure of the gp32–ssDNA filament.

While much of gp32 function (e.g. concentration regulation, efficient coating of ssDNA, and disruption of secondary structures) can be understood in terms of its relative binding affinities for different nucleic acid substrates (e.g. ssDNA, dsDNA and RNA) (8,22), the kinetic aspects of gp32 binding/dissociation are also important in understanding its other functions within the T4 system. gp32’s role in rapid and efficient replication of the T4 genome is one such example. Movement of the replication fork occurs at a rate of 400–700 bp/s *in vivo* (23), and this rate is presumably highly sensitive to, if not limited by, the kinetics of gp32 binding and dissociation. Prior work on gp32 kinetics showed that at cellular protein concentrations (∼2–3 μM), gp32 rapidly associates with ssDNA at an approximate rate of 15–20 s^−1^ (24,25). However, while gp32’s high binding affinity allows it to quickly coat and protect regions of ssDNA exposed during replication, it could also prevent the protein from being easily displaced from the substrate as required for rapid strand synthesis. Using stopped-flow methods, Lohman demonstrated that gp32 primarily dissociates from the ends of the cooperative clusters but that the rate of unbinding is too slow to account for the observed rate of DNA synthesis (24,26). Species-specific interactions between gp32 and T4 polymerase which enhance replisome processivity have been reported (27,28). Thus, active displacement of gp32 by T4 polymerase from the end of the filament constitutes a possible mechanism for the rapid removal and recycling of gp32 during replication. However, because this mechanism has not been directly observed, dissociation of gp32 during replication remains an important, open question.

In an attempt to address these issues, we use optical tweezers and atomic force microscopy (AFM) to investigate the structure and binding dynamics of gp32 with long (∼8 knt) ssDNA substrates, which, given the gp32 occluded site size of 7 nt, can accommodate ∼1000 proteins, allowing us to probe the large-scale, collective behavior of a many-protein system. Through DNA stretching and constant force techniques, we measure both the conformational changes of the ssDNA and the associated kinetics during gp32 binding and dissociation. Additionally, we compare these measurements with those performed with the noncooperative truncate, *II, in order to quantify the extent to which this behavior is driven by cooperative interactions. Our results show that cooperative binding of gp32 moderately reduces the contour length of ssDNA while drastically increasing its persistence length. Furthermore, we find that the gp32–ssDNA complex is highly dynamic. Under conditions of high protein concentration, gp32 is able to modulate its conformation on the DNA, resulting in an increase in the complex's contour length relative to its compacted state. This elongated conformation is unstable and marked by an additional phase of relatively rapid, noncooperative protein dissociation along the entire length of the ssDNA substrate.

## MATERIALS AND METHODS

### Purification of gp32

Full-length gp32 and its truncated form (*II) were prepared as previously described (29,30). Protein concentrations were determined spectrophotometrically using ϵ_280_^M^ = 3.7 × 10^4^ M^−1^ cm^−1^ (31).

### Optical tweezers system for measuring ssDNA conformation at constant force

**Figure 1.**
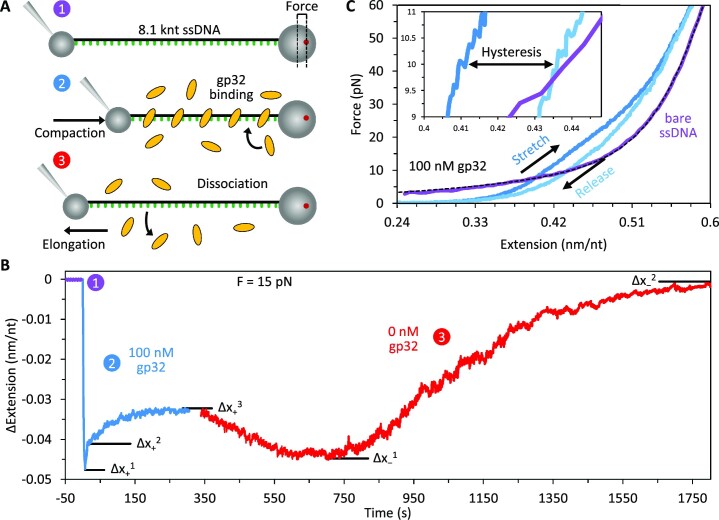
Measuring gp32 binding and ssDNA conformation. (**A**) An 8.1 knt ssDNA was tethered between two functionalized microbeads and extended until reaching a set tension as measured by beam deflection in the optical trap (1). The extension of the DNA molecule was continuously adjusted to maintain constant tension after introducing free protein (2) and after removing free protein (3) to measure gp32 binding and dissociation. (**B**) At a fixed force of 15 pN, the extension of ssDNA in the presence of 100 nM gp32 (blue) shows multiple binding phases with measured amplitudes of DNA extension change (Δx): an initial fast compaction (Δx_+_^1^) followed by two distinct elongation events with different kinetic rates (Δx_+_^2^ and Δx_+_^3^). Upon removal of free, unbound gp32 (red), two dissociation steps are observed. Initial dissociation results in recompaction of the substrate (Δx_−_^1^). Subsequent dissociation is marked by a slow increase in ssDNA extension as the DNA returns to its initial protein-free conformation (Δx_−_^2^ = 0). (**C**) The DNA was slowly (∼10 nm/s) stretched (blue) and released (light blue) in the presence of 100 nM gp32. At forces above ∼10 pN, gp32 compacts the DNA. Below ∼10 pN, the protein-DNA complex is elongated relative to bare ssDNA (purple with dashed black line showing fit to FJC). Notably, the release curve exhibits hysteresis (inset) between ∼5 and 30 pN tension.

An 8.1 knt ssDNA molecule tethered between a 2 μm anti-DIG and a 3 μm streptavidin functionalized bead (Figure 1A) was generated *in situ* by T7 exonuclease as described previously (32–34) and held at various fixed tensions. While dsDNA follows the extensible worm-like chain (WLC) polymer model (35,36), our ssDNA molecule is well fit by the freely jointed chain (FJC) (37), indicating that the formation of secondary structures due to sequence heterogeneity is negligible at forces ≥5 pN. Extension of the ssDNA was continuously altered to maintain the given force applied by the trapping laser in a binding buffer containing different fixed concentrations of gp32 diluted in 50 mM Na^+^ (45 mM NaCl and 5 mM NaOH), 10 mM HEPES at pH 7.5. Following incubation, we measured the dissociation of gp32 by replacing the protein-containing buffer with protein-free buffer. The extension of the ssDNA was controlled by a piezoelectric translational stage with 1 nm resolution, and the tension along the substrate was measured by laser deflection of the stationary optical trap (Figure 1A). Additionally, distance between the microbeads was measured using simultaneously recorded bright-field images to calculate the absolute ssDNA extension and correct for long-term thermal drift in the system. All data were analyzed using custom scripts in MATLAB (Mathworks) with uncertainty calculated as standard error of the mean (SEM) of three or more replicates (individual data points and specific numbers of replicates used for all average values are shown in corresponding supplementary figures).

### ssDNA stretching experiments and contour/persistence length measurements

In the presence of various gp32 concentrations, the ssDNA was slowly stretched at a rate of ∼10 nm/s to ensure equilibration at every force. The force-extension curve (FEC) of the ssDNA saturated with the noncooperative *II truncate (gp32 lacking its N-terminal domain) was fit with the FJC (37) up to 10 pN to compute the contour and persistence lengths of the complex. Following stretch and release, the FECs of the full-length gp32–ssDNA complex were fit with the WLC model (35,36) up to 5 pN to compute the contour and persistence lengths as functions of protein concentration. The FECs were binned with respect to force using a bin width of 1 pN, and the FEC of bare ssDNA was subtracted from these data to obtain the equilibrium change in ssDNA extension from both *II and wild-type (WT) gp32 binding. Uncertainties in average lengths and extension changes were calculated as the SEM of three or more replicate curves (numbers of replicates used for each condition are shown in Supplementary Figure S2).

### AFM imaging

M13mp18 phage vector DNA (7249 nt) was diluted to a concentration of 100 pM in a buffer of 150 mM Na^+^ (145 mM NaCl and 5 mM NaOH), 100 μM spermidine, 10 mM HEPES, pH 7.5 and incubated with gp32 (10, 100 or 1000 nM) at 37°C for 5 min. 5 μl of solution were deposited on a freshly cleaved mica surface. After 1 min, the sample was rinsed thoroughly with DI water and then air blown dry. The sample was imaged using peak force tapping mode with a MultiMode 8 AFM and Nanoscope V controller (Bruker) using tips with nominal width of 2 nm. Images were analyzed using custom MATLAB (MathWorks) scripts.

## RESULTS

Using optical tweezers, we observed the binding of gp32 to an 8.1 knt ssDNA molecule and obtained information about the dynamic structure of their complex through two sets of experiments (Figure 1). First, we measured the change in extension of the DNA substrate held under constant tension after introduction of fixed gp32 concentrations in buffer (Figure 1A). The exact response varies with respect to free protein concentration and DNA tension, both effects we analyze in detail below. In general, when the ssDNA is incubated with gp32 we observe up to three sequential steps of both DNA compaction and elongation (Δ*x*_+_^1–3^) before the protein–DNA complex equilibrates to a final extension (Figure 1B shows an example binding curve at 100 nM gp32, blue). Similarly, when free protein is replaced with protein-free buffer, multiple dissociation steps are observed (Figure 1B, red); the ssDNA initially recompacts (Δ*x*_−_^1^) before slowly extending (Δ*x*_−_^2^) toward its protein-free conformation. We have previously observed such multiphasic binding behavior with other single-stranded binding proteins (33,38). For analysis, we measured the amplitude and rate associated with each distinct step of DNA compaction/elongation.

Second, we slowly stretched the DNA (∼10 nm/s to maintain equilibration) to high force, more than doubling its end-to-end extension, in the presence of fixed gp32 concentrations. The measured force–extension curve (FEC) reveals structural details of the gp32–ssDNA complex which we analyze in detail below. For protein-free ssDNA (Figure 1C, purple), the flexible ssDNA is first straightened at low force, resulting in a large extension change over a small increase in force, before larger forces are required to elastically stretch the DNA backbone. The gp32–DNA complex in comparison (Figure 1C shows an example stretch curve at 100 nM gp32, blue), straightens at a lower force, reflecting rigidification of the ssDNA, but is more compact (shorter) than the protein-free DNA when the applied force exceeds ∼6 pN. Notably, upon reducing the substrate extension to release its tension (light blue) the complex is significantly more extended than during the initial stretch, indicating hysteresis in the restructuring of the gp32–ssDNA filament on the timescale of the stretch-release cycle (inset shows hysteresis at 10 pN).

### Binding dynamics of noncooperative *II truncate

**Figure 2.**
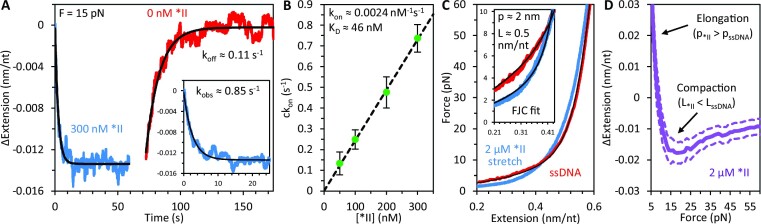
Binding dynamics of *II truncate. (**A**) The noncooperative *II gp32 truncate exhibits single-phase binding (blue, inset shows magnified exponential fit) with significantly reduced compaction relative to WT gp32. When free *II is removed (red) the ssDNA exponentially elongates back to its original length on a 10 s timescale, consistent with full dissociation of protein. (**B**) The measured rate of protein binding (*ck*_on_) is directly proportional to protein concentration and linearly fit to compute the concentration-independent bimolecular on-rate and *K*_D_ of *II at 15 pN. (**C**) When the ssDNA is slowly stretched (∼10 nm/s) in the presence of a saturating concentration (2 μM) of *II, the DNA is measurably shorter at high force (>10 pN) and longer at low force (<10 pN) due to changes in the contour and persistence lengths. The force-extension curve of the *II-saturated DNA was fit with the freely jointed chain (FJC) up to 10 pN (inset) to compute the contour (L) and persistence (p) lengths of the complex. (**D**) The average extension change of ssDNA as a result of *II binding is calculated at every force (1 pN increments) and plotted as a function of ssDNA tension (purple curve with dashed lines showing SEM).

To help separate the effects of cooperative filament formation from the initial binding of protein to the DNA substrate, we first characterized the binding and dissociation of the noncooperative truncate, *II, which lacks the N-terminal domain required for homotypic interprotein interactions. In contrast to the multiphasic binding of WT gp32, *II exhibits single-phased binding (Figure 2A shows an example binding curve at 300 nM *II, blue) well fit by a single observed rate constant (*k*_obs_). However, the final degree of compaction is significantly reduced relative to WT (maximal compaction for WT at 15 pN is ∼4-fold larger than *II), indicating that cooperatively-bound clusters are required for full DNA compaction. When free *II is removed (red) the ssDNA exponentially elongates back to its original length (*k*_off_ = 0.11 ± 0.01 s^−1^), consistent with full dissociation of protein. Assuming the rate of equilibration observed during incubation is the sum of the bimolecular rates of protein binding and dissociation (*k*_obs_ = *ck*_on_ + *k*_off_), we calculate the fundamental concentration-independent rate of free protein binding (0.0024 ± 0.0002 nM^−1^ s^−1^) (Figure 2B, Supplementary Figure S1). These rates imply that *II binds ssDNA in a simple on-off process with a dissociation constant, *K*_D_ = 46 ± 6 nM at 15 pN (Supplementary Table S1).

When the ssDNA is slowly stretched (∼10 nm/s) in the presence of a saturating concentration (2 μM) of *II, the presence of protein measurably shortens the DNA at high force (>10 pN) and lengthens it at low force (<10 pN), a consequence of changes in the contour and persistence lengths (Figure 2C). Because bare ssDNA is well modeled as a freely jointed chain (FJC) (a series of small rigid links that bend freely between segments), ssDNA saturated with a noncooperative protein, such as *II, in which the DNA remains flexible (freely jointed) between bound proteins, can also be modeled as an FJC. However, the length of each link is now determined by the protein's binding site size, rather the length of a single nucleotide, as it is the ssDNA-bound *II that now defines the inflexible subunit of the full polymer chain. We, therefore, fit the force-extension curve of the *II-saturated DNA with the FJC up to 10 pN (inset) to compute a contour length of 0.510 ± 0.008 nm/nt and a persistence length of 1.9 ± 0.1 nm, consistent with 2–3 nucleotide tight binding of the protein's binding site with the ssDNA (16). The relatively small persistence length, roughly spanning the protein's binding site size, indicates that the FJC model appropriately describes the *II–ssDNA complex. We note, however, that above ∼10–15 pN, the *II-saturated ssDNA curve deviates from the FJC, suggesting that the intrinsic polymer properties of the protein-DNA complex (e.g. its contour and persistence lengths) are sensitive to substrate tension. The average extension change of ssDNA as a result of *II binding is calculated at every force (1 pN increments) and plotted as a function of ssDNA tension (Figure 2D, purple curve with dashed lines showing SEM).

### Contour and persistence lengths of the gp32–ssDNA complex

**Figure 3.**
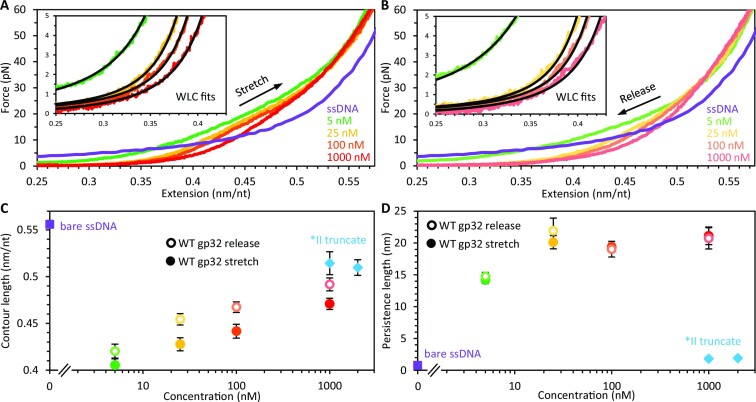
Contour and persistence lengths of gp32–ssDNA complexes. The ssDNA was slowly (∼10 nm/s) stretched (**A**) and released (**B**) in the presence of different concentrations of WT gp32. The protein–DNA complex becomes more extended as concentration is increased. In contrast to *II, the release curves exhibit an increase in extension (hysteresis, shown also in Figure 1C) relative to the initial stretch curves. The force-extension curves were fit with the WLC model up to 5 pN (insets) to compute the contour and persistence lengths of the complex. (**C**) The contour length reduction (relative to bare ssDNA, purple square) of WT is greater than *II (blue diamonds) but decreases with concentration during both stretch (filled circles) and release (empty circles). (**D**) Under the same conditions as shown in panel C, the WT complex exhibits a significantly greater persistence length than that of *II, plateauing to ∼20 nm at high protein concentration. The persistence lengths of the gp32–ssDNA complexes following release are nearly equivalent to those of the initial stretch.

We similarly measured stretch and release force-extension curves of ssDNA in the presence of different concentrations of WT gp32 (Figure 3A, B). In contrast to *II, the strong interprotein interactions of WT allow the protein to form long, flexible filaments along the DNA with persistence length similar to that of dsDNA (39). That is, the complex is not freely jointed, but rather a continuous, flexible polymer in which the subunits are more strongly correlated. We, therefore, fit the FECs of the WT complex with the worm-like chain (WLC) model up to 5 pN (insets) to compute the gp32–ssDNA contour and persistence lengths as functions of free protein concentration (Table S2). The protein–DNA complex becomes more extended with concentration; however, at forces above ∼10 pN the complex remains compacted relative to bare ssDNA. This compaction likely reflects helical winding of the DNA around the protein filament as modeled by van Amerongen et al. and others (40–44) which results in a significant reduction of the ssDNA contour length (Figure 3C, Supplementary Figure S2A). The observed lengthening at higher gp32 concentrations is coincident with an increase in the contour length of the complex relative to its compacted state. Additionally, we observe a moderate increase in the gp32–DNA persistence length with increasing gp32 concentrations up to 25 nM (Figure 3D, Supplementary Figure S2B). However, at gp32 concentrations ≥25 nM, sufficient to fully saturate the ssDNA, the persistence length plateaus at ∼20 nm, in reasonable agreement with previous light scattering experiments (39). This suggests that the extension increase seen at concentrations above 25 nM is primarily due to an increase in the contour length of the protein–DNA complex relative to its compacted state. This contour length increase continues without abatement up through our highest measured gp32 concentration (1 μM). At μM concentrations the protein begins to aggregate in solution (45–47), complicating the analysis of its ssDNA binding. Nevertheless, it appears that at concentrations greatly exceeding those required to saturate the DNA, additional proteins continue to bind and restructure the substrate. We therefore hypothesize that these additional gp32 proteins bind the saturated gp32 filament in a different mode with much higher *K*_D_. The observed DNA elongation upon additional gp32 binding may reflect partial unwinding of these helically compacted structures, allowing the protein-DNA complex to adopt a more extended conformation.

In contrast to *II, the release curves of WT gp32 exhibit an increase in extension relative to the initial stretch curves (hysteresis in Figure 1C), suggesting gp32 filament rearrangements induced by high force that do not have time to fully relax upon complex release. WLC fits to these release curves show that, relative to the properties of the initial stretch (filled circles), at protein concentrations ≥25 nM the DNA contour length is increased (Figure 3C, Supplementary Figure S2A), while the persistence length (Figure 3D, Supplementary Figure S2B, empty circles) remains unchanged. Thus, similar to the concentration-dependent behavior, the shifts in extension seen during release must be primarily driven by changes in the contour length of the gp32–DNA structure. We further probed this hysteresis by performing a ‘force switch’ experiment (Fig. S3) in which the ssDNA was initially incubated with 100 nM gp32 at a fixed force of 10 pN. Following equilibration, the tension on the DNA was increased to 50 pN for ∼100 s. When the force was lowered back to 10 pN, the complex exhibited a phase of exponential compaction (τ ∼ 50 s), re-equilibrating to an extension slightly longer than that observed prior to the increase in force. This compaction phase implies a tension-induced change in the conformation of the protein–DNA complex which is largely reversed upon relaxing the substrate back to lower tension. However, the observed increase in equilibrium extension following release suggests that these changes to the filament structure may partially persist over longer (>100 s) timescales.

**Figure 4.**
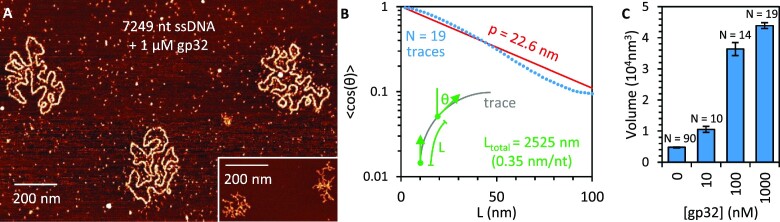
AFM imaging of gp32–ssDNA complex. (**A**) AFM image of 7249 nt long ssDNA incubated with 1 μM gp32. While protein-free ssDNA (inset, same scale) is condensed due to its tendency to fold back on itself, a result of its short persistence length and the formation of secondary structure formed between complementary bases in different regions of the ssDNA, the protein-saturated ssDNA forms one long continuous filament that can be traced along the 2D surface. (**B**) Traces of individual molecules are used to measure the average value of the cosine of the change in orientation angle (θ) between any two points separated by a length (*L*) along the trace. Average cos(θ) decreases exponentially as L increases, consistent with the WLC model. Fitting this decay parameter yields an effective persistence length (red line). (**C**) The total integrated volume of ssDNA molecules incubated with varying concentrations of gp32 is measured as a proxy for total protein bound to the substrate. At high concentration, the volume increases nearly 10× as compared to protein-free ssDNA, indicating the ssDNA–gp32 complex is protein-saturated.

We supplemented our optical tweezers measurements with AFM imaging of the gp32–ssDNA complexes. AFM imaging reveals the spatial position of the entire ssDNA substrate, rather than just its end-to-end extension, allowing direct measurement of gp32–ssDNA complex structure (Figure 4). A solution of ssDNA (100 pM) was incubated at 37°C for 5 min with a fixed concentration of gp32 (10, 100 and 1000 nM) in a 150 mM Na^+^ buffer containing 100 μM spermidine to stabilize binding of the ssDNA to the mica surface on which it was deposited (48). Note, the concentration of spermidine is more than three orders of magnitude more dilute than the Na^+^ concentration, and thus does not contribute significantly to gp32 binding. However, the increase in Na^+^ relative to our optical tweezers experiments may alter (reduce) the affinity of the protein for the DNA, requiring higher gp32 concentrations to fully saturate the substrate. While protein-free ssDNA is very flexible and able to form secondary structures with itself, resulting in a condensed structure that folds back on itself many times, ssDNA incubated with saturating quantities of gp32 appears as a linear polymer with limited flexibility (Figure 4A), enabling tracing of the ssDNA backbone. This trace permits analysis of the gp32–ssDNA complex's polymer properties of contour length and persistence length, as has been previously observed for dsDNA (49) (Supplementary Figure S4). According to the WLC polymer model, the orientation of two segments along the DNA backbone should be aligned with one another if the distance between them is much less than the persistence length, and uncorrelated if the distance between them is much greater than the persistence length. The exact decay in alignment for a two-dimensional WLC, which scales with the persistence length p, is written:


(1) \begin{equation*}\langle {\rm cos}(\theta )\rangle {\mathrm{ }} = {\mathrm{ }}{{\rm e}^{ - L/2p}}\end{equation*}

Here, θ is the difference in angular orientation for two points separated by a distance *L* along the polymer trace (49) (Figure 4B). Thus, for points separated by less than the persistence length, the change in angle is small, and for points separated by more than the persistence length, the orientations become progressively less coupled. We calculated the expected value of cos(θ) for any two points separated by distance *L* along the trace for each observed ssDNA molecule, and then for each value of *L*, averaged over all molecules observed. We do observe an exponential drop in orientation as *L* increases (Figure 4B, blue points), which is fit by Eq. (1) with fitting parameter *p* (red line). For the highest concentration of gp32 used (1 μM, 1:0.7 gp32 protein to ssDNA nt ratio), we measure a total contour length of 2525 ± 28 nm (0.348 ± 0.004 nm/nt) and persistence length 22.6 ± 1.1 nm in good agreement with our tweezer data. Note, DNA tends to form loops in solution, in which the strand crosses over itself forming a contact, that can be preserved during the deposition process. This results in an orientation anticorrelation between points on either side of the loop, a feature not produced by a random polymer model. Thus, while we use the entirety of each DNA molecule trace to measure the total contour length, we measure persistence length using only the longest segment of each DNA molecule that is loop free. We confirmed that this analysis method properly measures both values using dsDNA as a control (Supplementary Figure S4). For 100 nM gp32 concentration (1:7 gp32 protein to ssDNA nt ratio), we measure a slightly reduced contour length 2490 ± 56 nm (0.343 ± 0.008 nm/nt) and persistence length 20.3 ± 0.8 nm. In contrast, we found that 10 nM gp32 (1:70 gp32 protein to ssDNA nt ratio) was insufficient to linearize the ssDNA, such that the molecules could not be traced (Supplementary Figure S5). These results are in agreement with the previously measured binding site of ∼7 nt for gp32 on ssDNA (9).

We also measured the binding of gp32 to the ssDNA using volumetric analysis (Figure 4C). That is, for each observed ssDNA molecule we integrated over the height above background for each pixel in the image to measure the effective volume of the molecule as measured by the AFM tip. While the absolute value of this volume measurement depends on many factors external to the molecule of interest such as AFM tip size, it has been previously shown that the volume of proteins can be effectively compared to DNA substrates and that measured volume scales linearly with molecular mass (50). For reference, our system (2 nm nominal tip size) measures a single 7.25 knt ssDNA molecule (2.4 MDa) to have an integrated volume of approximately 5000 nm^3^. When incubated with 10 nM gp32, this volume is roughly doubled, indicating measurable protein binding, even if the amount of protein bound is insufficient to filament the ssDNA. Saturating concentrations of gp32 increase the measured volume by an order of magnitude, suggesting the gp32–ssDNA complex contains ∼10X more protein than ssDNA by volume/mass, in reasonable agreement with a binding site size of 7 nt (∼1000 per ssDNA molecule) and protein molecular mass of 33.5 kDa per gp32 protein. Overall, these AFM measurements support our optical tweezers measurements showing moderately reduced contour length and drastically increased persistence length for the gp32–ssDNA complex. Additionally, while 100 nM gp32 is sufficient to filament and linearize the entirety of the ssDNA, we measure a slight increase in total protein bound (via integrated volume), contour length, and persistence length when the gp32 concentration is increased to 1 μM. We also calculate the radius of the gp32–ssDNA filament through comparison to the well-defined dimensions of dsDNA. Both of these structures can be geometrically approximated as long flexible cylinders with total volume equal to their length multiplied by cross-sectional area. Thus, the ratio of the measured volumes of dsDNA and the same length of ssDNA saturated with gp32 is expressed:


(2) \begin{equation*}\frac{{{V_{ds}}}}{{{V_{ss}}}} = \frac{{{L_{ds}}\pi r_{ds}^2}}{{{L_{ss}}\pi r_{ss}^2}}\end{equation*}

\begin{equation*}r_{ss}^{} = \sqrt {\frac{{{V_{ss}}{L_{ds}}}}{{{V_{ds}}{L_{ss}}}}} r_{ds}^{}\quad\end{equation*}

Our length and volume measurements made by AFM combined with the known radius of the dsDNA helix of approximately 1 nm, give a calculated radius of the gp32–ssDNA filament of 2.1 ± 0.1 nm, in good agreement with previous measurements of gp32–ssDNA structure (40,51).

### Force dependence of gp32 binding

**Figure 5.**
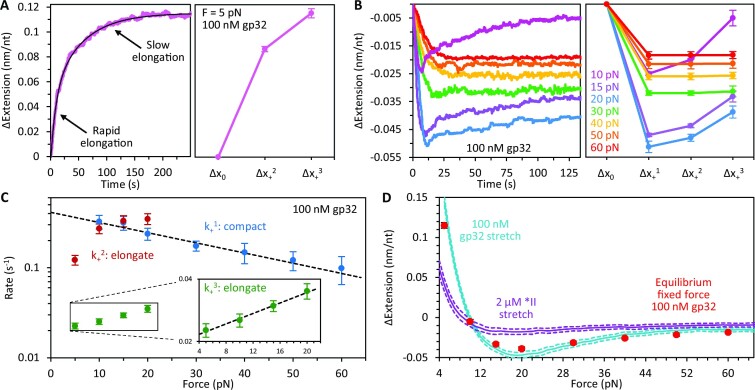
Force dependence of gp32 binding. (**A**) Representative curve (left) and average extension changes (right) associated with binding of 100 nM gp32 at 5 pN. At this force, no gp32-mediated compaction is observed (Δ*x*_+_^1^ = 0). Instead, the DNA is immediately elongated in two kinetically distinct phases: an initial rapid elongation (Δ*x*_+_^2^) followed by a slower elongation that equilibrates to a final extension (Δ*x*_+_^3^). The curves are fit with a two-rate decaying exponential function to extract the rates and amplitudes associated with both phases of ssDNA elongation. (**B**) Representative curves (left) and average extension changes (right) associated with binding of 100 nM gp32 as a function of tension. At forces ≥10 pN, gp32 compacts the ssDNA. Between 10 and 20 pN the DNA compaction increases with tension and the extension of the substrate exhibits multiple phases during protein binding: an initial compaction (Δ*x*_+_^1^) followed by two partial elongation events (Δ*x*_+_^2^ and Δ*x*_+_^3^). Following compaction, the curves are fit with a two-rate decaying exponential to extract the amplitude and rate associated with each phase of elongation. Further increase in tension results in a single-phased extension reduction that decreases with force. (**C**) The rate of each binding phase is calculated as a function of ssDNA tension. The initial compaction rate (*k*_+_^1^, blue) decreases exponentially with force (fit shown as dashed black line). The rate of rapid elongation (*k*_+_^2^, red) increases with tension and approaches the low force compaction rate. The secondary elongation rate (*k*_+_^3^, green) is significantly slower but increases exponentially with tension (fit shown as dashed black line in inset with log-linear scale). (**D**) The average ssDNA extension change as a result of gp32 binding is calculated at every force (1 pN increments, blue curve with dashed lines showing SEM) and compared with the extension change of ssDNA saturated with *II (purple curve, replotted from Figure 2D). WT extension changes are consistent with equilibrium extension changes from constant force measurements (red circles).

We probed the force dependence of WT gp32 binding by measuring the temporal change in extension of the ssDNA in the presence of 100 nM gp32. At 5 pN (Figure 5A, Supplementary Figure S6A, Supplementary Table S3), gp32 elongates the ssDNA in two distinct phases (no initial compaction is observed): an initial rapid elongation, Δ*x*_+_^2^, followed by a slower elongation that equilibrates to a final extension, Δ*x*_+_^3^. Total elongation at low tension must be partially driven by the large (∼30-fold) increase in the persistence length of the ssDNA (Figure 3). In contrast, at forces ≥10 pN (Figure 5B, Supplementary Figure S6B), gp32 compacts the DNA, indicating that at moderate and high force the reduction in contour length outcompetes the persistence length increase to drive overall compaction of the ssDNA. Between 10 and 20 pN the DNA compaction increases with tension and the extension of the substrate exhibits multiple phases during protein binding: an initial compaction (Δ*x*_+_^1^) followed by two partial elongation events (Δ*x*_+_^2^ and Δ*x*_+_^3^). Further increase in tension results in a single-phased extension reduction that decreases with force. Thus, the degree of ssDNA compaction driven by gp32 filamentation is highly sensitive to substrate tension, with high force disfavoring the highly compacted protein–DNA state.

The kinetics associated with each binding phase were evaluated by measuring the rate at which the ssDNA extension approached Δ*x*_+_^1^ (*k*_+_^1^), as well as the transition rates from Δ*x*_+_^1^ to Δ*x*_+_^2^ (*k*_+_^2^) and Δ*x*_+_^2^ to Δ*x*_+_^3^ (*k*_+_^3^, Figure 5C, Supplementary Figure S6C, Supplementary Table S3). The initial rate, *k*_+_^1^ (blue), decreases exponentially with tension as the applied force opposes the gp32-mediated DNA compaction, exhibiting a ∼3-fold reduction between 10 and 60 pN. This force dependence likely arises from a characteristic length change (Δ*x*) associated with each protein binding event which, when multiplied by the applied force, presents an energy barrier that modulates the force-dependent rate as:


(3) \begin{equation*}k(F) = {k_0}{e^{F \cdot \Delta x/{k_B}T}}\end{equation*}

Fitting the compaction rate with a single decaying exponential (dashed line) yields a length change of −0.105 ± 0.014 nm associated with gp32 binding. The rate of subsequent fast elongation, *k*_+_^2^ (red), initially increases rapidly with tension but asymptotes to the DNA compaction rate (*k*_+_^1^) seen at low force. The secondary elongation rate, *k*_+_^3^ (green), is significantly slower than the preceding transitions but increases exponentially with force (fit shown as dashed line in inset), giving an approximate length change of 0.112 ± 0.014 nm associated with filament unwinding.

The average equilibrium extension change of ssDNA as a result of gp32 binding was calculated at every force (1 pN increments) and plotted as a function of DNA tension (Figure 5D, blue curve with dashed lines showing SEM). These extension changes (calculated by slowly stretching the ssDNA in the presence of 100 nM gp32, Figure 3A) are consistent with the equilibrium extension changes from constant force measurements (red circles) indicating that the protein-DNA complex is in equilibrium throughout stretching. Comparing the changes in extension to those of *II (purple curve, replotted from Figure 2D) reveals that at low force the elongation of the DNA is significantly greater for WT gp32, a consequence of its increased persistence length (Figure 3D). At higher forces, the gp32–ssDNA complex becomes considerably more compact due to the reduction in contour length from cooperative protein filamentation (Figure 3C). At very high force, however, the extension change of the gp32 complex approaches that of *II, suggesting that tension may inhibit cooperative protein-protein interactions required for DNA compaction.

### Force dependence of gp32 dissociation

**Figure 6.**
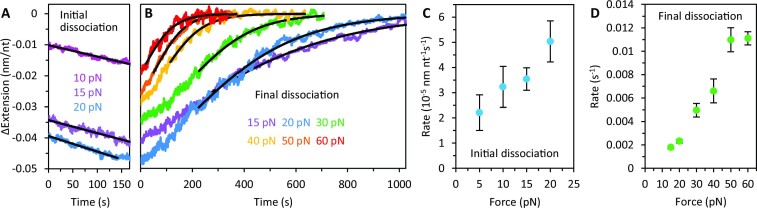
Force dependence of gp32 dissociation. Representative dissociation curves following incubation with 100 nM gp32. Note, representative initial and final dissociation curves are shown as separate panels for clarity (see Figure 1B for full trace of gp32 dissociation). (**A**) Upon removal of free protein, initial dissociation of gp32 leads to recompaction of the ssDNA at forces ≤20 pN. Recompaction of the DNA is linear in time and fit with a straight line to compute an initial dissociation rate. (**B**) Further dissociation, occurring after recompaction, is marked by an increase in extension as the DNA returns to its protein-free conformation (*t* = 0 s corresponds to the beginning of the elongation phase, occurring ∼200 s after free protein is removed). While at high tensions, dissociation is characterized by a single exponential, lower tensions result in an initial near linear elongation (up to ∼200 s), before exponentially decaying to the ssDNA’s protein-free extension. The curves are fit with a single decaying exponential following the linear phase of elongation (i.e. upon initial decay of the DNA elongation phase) to approximate a final dissociation rate. Both the initial (**C**) and final (**D**) dissociation rates increase with tension.

Upon removal of free protein, initial dissociation of gp32 leads to recompaction of ssDNA at forces ≤20 pN (Figure 6A). Substrate recompaction is linear in time, rather than exponential, and fit with a straight line to compute the initial dissociation rate. The linearity of this dissociation phase indicates a zeroth-order reaction, suggesting: (i) gp32 primarily unbinds from the ssDNA at the ends of the cooperative protein clusters, consistent with previous dissociation measurements (26) and (ii) the number of cluster ends remains relatively small and approximately constant during this initial step; that is, the protein clusters do not substantially redistribute during this process. Recompaction of the complex upon such dissociation is likely associated with rewinding of the released ssDNA on the remaining gp32 filaments, shortening the length of the complex. Eventually, the DNA substrate begins to elongate and approach its protein-free length, indicating full (final) protein dissociation (Figure 6B). At lower forces, the extension change during final dissociation again initially proceeds linearly before exponentially decaying to zero. In contrast, at high tensions, gp32 dissociation can be characterized by a single exponential rate, reflecting progressive disruption of the gp32–gp32 interactions at higher force. The curves are all fit with a single decaying exponential, discarding the initial linear region where present. Both the initial and final dissociation rates strongly increase with tension indicating that gp32 binding stability and cooperativity decreases with force (Figure 6C, D, Supplementary Figure S7, Supplementary Table S4), and that the transition state for both steps of dissociation lies much closer to the unwound and unbound state than to the wound and bound state, *i.e*. significant ssDNA unwinding is required for gp32 unbinding. However, the high force dissociation rate of WT (∼0.01 s^−1^, Figure 6D) is ∼10-fold lower than that of *II at 15 pN (Figure 2A), suggesting that, while protein-protein interactions may be constrained by tension, gp32 remains moderately cooperative such that dissociation may still be dominated by cluster ends. Moreover, under low salt conditions (similar to those used here), previous dissociation studies reported a two-step dissociation process wherein gp32 dissociates first by sliding off the end of a protein cluster to form a noncooperatively-bound intermediate which subsequently unbinds from the DNA (24). The much slower rate of WT dissociation relative to noncooperative *II implies that the rate of unbinding observed within our experiments primarily reflects a timescale of cooperatively-bound proteins breaking their gp32–gp32 interaction and sliding away from the cluster (i.e. a rate of de-polymerization).

We additionally probed gp32 dissociation through a series of ‘force jumps’ (Fig. S3C). The ssDNA was initially incubated with 100 nM gp32 at a fixed force of 15 pN. Following equilibration, the tension on the DNA was increased to 50 pN for ∼20 s. The tension was then lowered back to 15 pN where the complex experienced a phase of exponential compaction as it equilibrated to a more stable conformation. Free protein was replaced with protein-free buffer leading to further (linear) DNA compaction as gp32 dissociated from the ends of the protein clusters. The tension on the substrate was subsequently cycled between 15 and 50 pN repeatedly. During cycling the ssDNA extension at 15 pN exhibited a biphasic profile, compacting further before extending towards its protein-free conformation, while at 50 pN dissociation was single-phased, consistent with our constant force measurements (Figure 6A, B).

### Concentration dependence of gp32 binding

**Figure 7.**
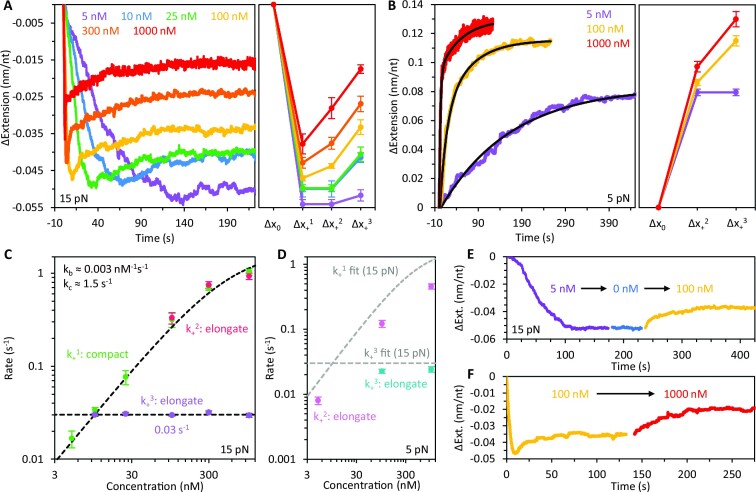
Concentration dependence of gp32 binding. (**A**) Representative curves (left) and average extension changes (right) associated with binding of gp32 as a function of free protein concentration at 15 pN. Both the maximum initial compaction (Δ*x*_+_^1^) and the equilibrium extension reduction of the ssDNA decrease with protein concentration. Rapid elongation (Δ*x*_+_^2^) is only observed at concentrations ≥100 nM. Additionally, slow partial elongation of the DNA (Δ*x*_+_^3^) vanishes at 5 nM. Under the conditions in which we observe biphasic elongation, the curves are fit with a two-rate decaying exponential to extract the rates and amplitudes of those phases. (**B**) Representative curves (left) and average extension changes (right) associated with the binding of gp32 as a function of concentration at 5 pN. The elongation of the ssDNA is biphasic at concentrations ≥100 nM, marked by an initial rapid increase in DNA extension (Δ*x*_+_^2^) which is followed by a slower elongation event that equilibrates to a final extension (Δ*x*_+_^3^). Both the transient elongation and the equilibrium extension of the ssDNA increase with protein concentration. (**C**) The rate of each binding phase is calculated as a function of gp32 concentration at 15 pN. The rate of compaction (k_+_^1^, green) initially increases linearly with concentration before approaching an asymptote at high protein concentration. The rate of rapid elongation (*k*_+_^2^, red) increases with concentration and is approximately equivalent to the initial compaction rate. The slow elongation step (*k*_+_^3^, purple), however, is independent of the free protein concentration. (**D**) The rate of each binding phase is calculated as a function of gp32 concentration at 5 pN. The initial elongation of the DNA (*k*_+_^2^, pink) increases with concentration. However, this rate is ∼2-fold slower than at 15 pN (fit line from C replotted in grey for comparison). The secondary elongation step (*k*_+_^3^, blue) is slightly slower than that measured at 15 pN (fit line from C replotted in grey for comparison). (**E**) ssDNA compaction was monitored during sequential changes in protein concentration. In the presence of 5 nM gp32 (purple), the ssDNA exhibits compaction without subsequent elongation. When free protein is rinsed out (blue) and replaced with 100 nM gp32 (yellow), the extension increases and equilibrates to a length consistent with that observed when the DNA is incubated directly with 100 nM (panel A, yellow). (**F**) When the concentration is switched from 100 nM (yellow) to 1000 nM (red), the complex equilibrates to an extension consistent with that observed when the DNA is incubated directly with 1000 nM (panel A, red).

We measured the concentration dependence of gp32 binding at both low (5 pN) and high force (15 pN, Figure 7, Supplementary Table S5). At 15 pN, both the transient compaction (Δ*x*_+_^1^) and equilibrium compaction (Δ*x*_+_^3^) of ssDNA decrease with protein concentration (Figure 7A, Supplementary Figure S8A). Following the initial relatively rapid compaction step (Δ*x*_+_^1^), the subsequent fast elongation phase (Δ*x*_+_^2^) is only observed at gp32 concentrations ≥100 nM but becomes considerably more pronounced above 300 nM. Additionally, the slow partial elongation (Δ*x*_+_^3^) vanishes at 5 nM as the DNA extension change becomes single-phased. Measurements at 5 pN show that the complex is elongated relative to bare ssDNA (Figure 7B, Supplementary Figure S8B), a consequence of its increased persistence length. Similar to the behavior at 15 pN, the elongation of the ssDNA is biphasic at concentrations ≥100 nM, marked by an initial rapid increase in DNA extension (Δ*x*_+_^2^), which is followed by a slower elongation event that equilibrates to a final extension, Δ*x*_+_^3^.

Consistent with the DNA stretching curves shown in Figure 3, the equilibrium extension of the complex increases with concentration at both low and high force. These stretching data also revealed a continual increase in the protein–DNA contour length with respect to concentration, consistent with the changes in equilibrium extension seen at constant force (Figure 7A, B). Thus, elongation of the DNA following initial protein binding is associated with an increase in the contour length of the gp32–ssDNA complex. These elongation events likely reflect unwinding of the helical protein filaments along the ssDNA. Moreover, the observed concentration dependence suggests that these conformational changes are concomitant with additional protein binding, and thus facilitated by an increase in protein density along the DNA.

The rate of each binding phase was calculated as a function of gp32 concentration at 15 pN (Figure 7C, Supplementary Figure S8C, Supplementary Table S5). The rate of compaction, *k*_+_^1^ (green), initially increases linearly with concentration but begins to asymptote at high protein concentration. Previous studies on gp32 binding revealed that gp32 initially binds ssDNA as a monomer prior to forming cooperative clusters along the lattice (25). Furthermore, we’ve shown that the formation of gp32 oligomers drives additional substrate compaction by further reducing the contour length of the DNA molecule. Thus, we fit *k*_+_^1^ with a two-step reaction model allowing us to deconvolve the protein's diffusion-limited bimolecular binding rate, k_b_, from the rate of subsequent compaction, *k*_c_, due to initial cluster formation. We measure a protein on-rate of 0.003 nM^−1^ s^−1^, in good agreement with the on-rate of the noncooperative truncate *II, as well as the compacting rate due to initial gp32 oligomerization, *k*_c_ = 1.5 s^−1^. Using the on-rate and the approximate dissociation rate calculated previously (Figure 6D, 15 pN), we estimate a *K*_D_ of ∼1 nM at 15 pN.

The rate of rapid elongation, *k*_+_^2^ (Figure 7C, red), increases with concentration and agrees with the initial compaction rate suggesting that this phase of elongation may reflect a similar process of diffusion-limited protein binding. The slow elongation phase, *k*_+_^3^ (purple), however, is independent of the free protein concentration, indicating an additional rate-limiting step which may reflect slow reorganization of the gp32 clusters to accommodate binding of additional proteins that subsequently unwind the filament. While both phases of elongation are coincident with increases in the protein-DNA contour length, the difference in rates associated with these events suggests distinct mechanisms of ssDNA binding. Given that the rapid elongation phase is only present at very high protein concentrations (when the binding rate is on the same order as the rate of initial cluster formation), we hypothesize that this step occurs prior to complete filamentation of the gp32 along the DNA, allowing the proteins to bind relatively quickly. Once the protein-protein interactions are fully established, further protein binding becomes significantly slower and rate-limited by restructuring of the complex as gp32 must bind into the existing cooperative filament. The rate of this reorganization step may reflect a timescale of breaking the gp32–gp32 contacts in order to accommodate the new protein.

In addition to our high force binding measurements, the rate of each binding phase was calculated as a function of gp32 concentration at 5 pN for comparison (Figure 7D, Supplementary Figure S8D, Supplementary Table S6). Similar to the behavior at 15 pN, the initial elongation of the DNA, *k*_+_^2^ (pink), increases with concentration. However, this rate is ∼2-fold slower than at 15 pN (fit line from C is replotted for comparison), consistent with the force-dependent analysis shown in Figure 5. Likewise, the secondary elongation step, *k*_+_^3^ (teal), is slightly slower than that measured at 15 pN (fit line from C is replotted for comparison) but appears to be similarly concentration-independent.

To further probe the concentration dependence of the gp32–ssDNA conformational dynamics we performed ‘concentration switch’ experiments (Figure 7E, F) wherein ssDNA compaction/elongation was monitored during sequential changes in protein concentration. In the presence of 5 nM gp32 (purple), the ssDNA exhibits compaction without subsequent relaxation (Figure 7E). When free protein is rinsed out (blue) and replaced with 100 nM gp32 (yellow), the extension increases and equilibrates to a length consistent with that observed when the DNA is incubated directly with 100 nM (panel A, yellow). Furthermore, when the concentration is switched from 100 nM (yellow) to 1000 nM (Figure 7F, red), the complex equilibrates to an extension consistent with that observed when the DNA is incubated directly with 1000 nM (panel A, red). Thus, with respect to protein concentration, the final equilibrium state of the gp32-DNA complex appears to be largely path independent. Additionally, the observed binding modes are likely facilitated by further protein binding, consistent with the idea that these conformational transitions are driven by changes in the protein density along the substrate.

### Concentration dependence of gp32 dissociation

**Figure 8.**
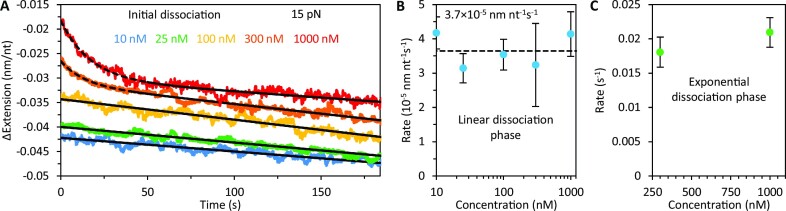
Concentration dependence of gp32 dissociation. (**A**) Representative curves associated with the initial dissociation phase of gp32. At protein incubation concentrations ≤100 nM recompaction of the ssDNA during initial dissociation is strictly linear in time. At high protein concentrations (≥300 nM) the DNA exhibits two phases of recompaction: an initial rapid exponential recompaction followed by a slower linear recompaction. The curves are fit with the sum of a linear and single decaying exponential function to extract the rates of both compaction phases. Both the linear (**B**) and fast exponential (**C**) dissociation rates are largely insensitive to initial protein incubation concentration.

As we have shown, initial dissociation of gp32 leads to recompaction of the DNA at forces ≤20 pN. We probed this recompaction phase as a function of protein incubation concentration while the DNA was held under 15 pN tension (Figure 8, Supplementary Table S7). At concentrations ≤100 nM, recompaction of the ssDNA is strictly linear in time (fits shown as solid lines), consistent with dissociation occurring from the ends of the cooperative protein clusters. Notably, however, at higher protein concentrations (≥300 nM) the DNA exhibits two phases of recompaction: an initial rapid recompaction, occurring exponentially, followed by a slower linear recompaction similar to the one observed at lower [gp32]. Appearance of a rapid compaction phase (fits shown as dashed lines) suggests that high concentrations of free protein give rise to an additional protein-DNA binding mode that is significantly less stable and noncooperative. We find that, while the equilibrium complex length is strongly concentration-dependent, both the linear (Figure 8B, Supplementary Figure S9A) and exponential (Figure 8C, Supplementary Figure S9B) dissociation rates are largely insensitive to the initial protein incubation concentration. The exponential nature of the rapid dissociation step indicates that gp32 unbinding during this phase occurs across the entire strand rather than strictly from the cluster ends. Moreover, this initial dissociation step is ∼10-fold faster than the subsequent final dissociation at 15 pN (Figure 6B), i.e. gp32 dissociation during this initial phase is clearly facilitated by substrate overcrowding.

While we were unable to explore the fast protein dissociation at [gp32] > 1 μM due to gp32’s tendency to form protein aggregates in solution (45–47), our available data allows us to hypothesize that the fast dissociation mode sets in when the gp32 density on ssDNA exceeds some critical level. Based on the data in Figure 8A it seems plausible that when the complex compaction per nt due to gp32 binding becomes smaller than ∼0.03 nm/nt, fast dissociation sets in. The rate of fast g32 dissociation becomes only slightly faster with increasing [gp32] (Figure 8C), but the fraction of fast dissociating protein increases significantly with [gp32], most likely reflecting the fact that any filament oversaturation is always relieved to the same critical level as the filament becomes more stable.

## DISCUSSION

### Dynamic binding modes of gp32

Our stretching curves revealed a protein-DNA complex that is far more dynamic than previously thought. Under conditions of high protein concentration the DNA became oversaturated with gp32, resulting in a protein-DNA contour length significantly longer than that seen at much lower, albeit saturating, protein concentrations (see Figure 3). To explore the kinetics of gp32 binding and dissociation we performed gp32 ‘concentration switch’ experiments while holding the ssDNA template at various constant forces (see Figures 5–8). We observed multistep gp32 binding kinetics that involved initial fast bimolecular binding to unsaturated ssDNA with the rate ∼0.003 nM^−1^s^−1^, similar to the binding of the noncooperative truncate *II. This rate appears to be only slightly affected by the force on ssDNA, suggesting that minimal DNA shrinking is required at initial binding before transitioning into the filamented state. At moderate forces, initial protein filamentation subsequent to gp32 binding resulted in significant DNA compaction not observed with *II. This fast compaction was followed by partial elongation of the complex, likely reflecting unwinding of gp32 filaments to accommodate additional protein. As the final equilibrium filament length increased with [gp32], this process clearly involved further gp32 binding, likely rate-limited by protein-protein unbinding required to accommodate new protein in the filament. This process was also only weakly force-dependent, suggesting that only a very small complex elongation is required to reach the transition state of this conformational change (Figure 5C). This likely means that the rate-limiting step of additional gp32 binding into the saturated filament involves unbinding of the existing short-range gp32–gp32 contacts within the filament. At higher ssDNA tension (≥ 30pN) the elongation steps were no longer observed, likely due to higher forces progressively disrupting the gp32-gp32 interactions required for full DNA compaction, resulting in a single-phased approach to a less compacted complex length (Figure 5B).

gp32 dissociation from the filament also occurs via several sequential processes. The first step appears to be slow gp32 dissociation from the ends of the few cooperative filament clusters, resulting in recompaction of the complex (Figure 6A). This compaction phase presumably reflects rewinding of the ssDNA, released during gp32 dissociation, on the remaining gp32–ssDNA filament. As this compaction happens in opposition to the DNA tension, it disappears at forces ≥30 pN. Once the maximum ssDNA winding within the filament is achieved, subsequent gp32 dissociation leads to ssDNA release from the filament followed by an increase in the extension of the complex (Figure 6B). The initial phase of this process is relatively slow, but becomes faster as more protein dissociates, creating new filament boundaries, eventually leading to exponential complex elongation at late times (ranging between 100 and 500 s, dependent on the stretching force), characterized by relatively slow rates (compared to noncooperative *II dissociation). Strong facilitation of the final gp32 dissociation phase by force implies that the final release of ssDNA has a transition step that requires significant DNA unwinding, consistent with gp32 dissociation from ssDNA in its maximally wound state. Eventually all gp32 dissociate, releasing bare ssDNA, as the extension of the complex approaches that of protein-free DNA.

### gp32-ssDNA filament structure

We showed that gp32 filament formation leads to a significant reduction in the contour length of the ssDNA which we observed as substrate compaction at forces ≥ 10 pN. Our stretching data revealed a much stronger equilibrium complex compaction by WT gp32 as compared to its noncooperative counterpart, *II. This implies that the strong ssDNA compaction by gp32 is related to its ability to form highly cooperative filaments that compact ssDNA by helically winding it around the protein, as was previously modeled by van Amerongen et al. and Scheerhagen *et al.* (40–44). Achieving a helical filament would only require that the interface of a contiguously bound protein be offset at a constant angle relative to its neighboring protein, and in the absence of a detailed crystal structure (e.g. multiple cooperatively-bound gp32 molecules crystalized in complex with ssDNA) this remains, perhaps, the most reasonable model.

The geometrical parameters of an ideal protein–DNA helical filament are related as follows:


(4) \begin{equation*}\frac{R}{\rho } = \frac{1}{{2\pi }}{\left[ {{{\left( {\frac{L}{{L^{\prime}}}} \right)}^2} - 1} \right]^{{1 \mathord{\left/ {\vphantom {1 2}} \right.} 2}}}\end{equation*}

**Figure 9.**
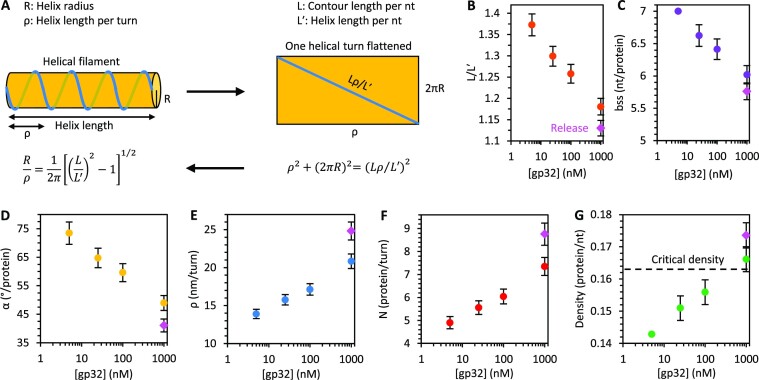
Geometric parameters of gp32–ssDNA helix. (**A**) Geometrical model of an ideal protein–DNA helix relating the ssDNA contour length (*L*), helix length (length along translational axis, *L*′), radius (*R*) and pitch (ρ). gp32–ssDNA helix parameters are calculated as functions of protein concentration using the measured contour length of bare ssDNA (*L* = 0.56 nm/nt), effective contour lengths of the protein–DNA complex (*L*′, Figure 3C), and helix radius (*R* = 2.1 nm – measured by AFM, see Figure 4 and its discussion in the main text). The ratio *L*/*L*′ (**B**), the protein binding site size (bss, **C**), and the twist angle between neighboring proteins (α, **D**) decrease with free protein concentration. The helical pitch (ρ, **E**), number of proteins per turn (*N*, **F**) and the protein density (**G**) increase with concentration. The helical parameters associated with the longest observed gp32-DNA contour length measured at 1 μM [gp32] during release (Figure 3C, open red circle) are indicated by a magenta diamond. The protein density at which we begin to observe the rapid exponential dissociation phase is indicated by a dashed line in (G).

Here, *R* is the helix radius, *ρ* is the helical pitch (i.e. length per turn), *L* is the contour length of the ssDNA per nt (0.56 nm/nt),and *L′* is the effective contour length of the DNA wound around the protein helical filament per nt (i.e. length of helix along the translational axis, see Figure 9A). Assuming an ideal helical structure with constant filament radius of *R* = 2.1 ± 0.1 nm, as suggested by our AFM imaging, the contour lengths of bare ssDNA and ssDNA saturated with gp32 (Figure 3) obtained from our stretching curves give a radius:pitch ratio of 0.15 ± 0.01. That is, the length per turn along the translational axis is about 7-fold larger than the radius of the helix. Here we have used the contour length of the complex at the lowest bulk gp32 concentrations of 5 nM (0.41 nm/nt), corresponding to its most compact state, and compared it to the contour length of bare ssDNA (0.56 nm/nt), yielding a ratio of *L*′/*L* = 0.73 ± 0.01. The corresponding helical pitch according to Eq. (4) is *ρ* = 13.9 ± 0.6 nm or ρ/*L*′ = 34 ± 2 nt of wound ssDNA per turn. Assuming that in this most relaxed and optimally wound filament state the gp32 binding site size on ssDNA is 7 nt, as measured previously (9), we estimate the length along the ssDNA per protein 0.56 nm/nt · 7 nt/protein = 3.9 ± 0.1 nm/protein and the number of proteins per helical turn, *N* = 34 nt/7 nt = 4.9 ± 0.3. In other words, the gp32 filament contains ∼5 proteins per turn with a twist angle of 360°/4.9 = 73 ± 4° between neighboring gp32 molecules. Furthermore, we can estimate the length of a single gp32 protein along the helical axis, h, for the most relaxed filament to be h = (*L′*/*L*) · 3.9 nm/protein = 2.8 ± 0.1 nm/protein.

**Figure 10.**
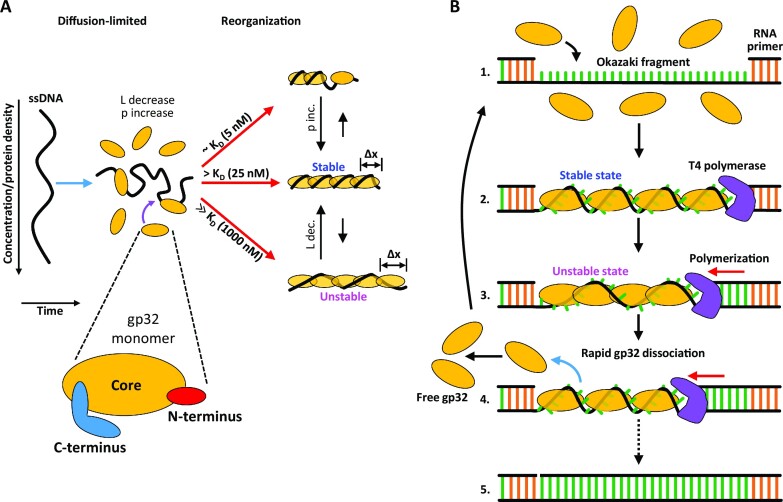
gp32 binding states and function. (**A**) Diagram illustrating the different concentration-dependent gp32 binding states and pathways measured in this study. gp32 binding reduces the contour length (*L*) and increases the persistence length (p) of ssDNA. At gp32 concentrations approximately equal to *K*_D_ (∼5 nM), gp32 filamentation along the ssDNA is incomplete. At [gp32] > *K*_D_ (∼25 nM), the DNA is optimally saturated and filamented with gp32, giving rise to an increase in persistence length as the complex reorganizes into its most stable conformation. At protein concentrations well above saturating (∼1000 nM), the protein density along the DNA increases further, resulting in an increase in the protein-DNA contour length as the complex equilibrates to a more extended (Δx) and less stable conformation. (**B**) Diagram illustrating a model for the function of gp32’s unstable binding mode during DNA replication. During lagging strand synthesis, Okazaki fragments are formed (1) and subsequently coated with gp32 in a stable binding conformation (2). Polymerization along the strand drives an increase in protein density as the ssDNA segment shortens, forcing the gp32 filament to adopt a less stable conformation (3) that results in rapid protein dissociation and recycling (4). This process continues until the lagging strand is completely synthesized (5).

As we titrated in more gp32 protein the equilibrium complex extension increased. Assuming the filament radius, *R* = 2.1 nm, as well as the length, *h* = 2.8 nm of each gp32 protein along the filament axis remain constant, the observed filament lengthening implies that more proteins join the filament and the protein binding site size, bss, on ssDNA shrinks according to the expression bss = *h*/*L′*. For example, as the apparent filament length along the axis (*L′*) approaches the contour length of bare ssDNA, i.e. *L′* → *L*, bss = 5 nt/protein. Furthermore, as more proteins join the filament its helical pitch increases continuously according to Eq. (4), and the number of proteins bound per turn, *N* = *ρ*/*h*, grows, while the twist angle per protein, α = 360°/*N* decreases. Strikingly, the extension of the complex continues without saturation up until our highest [gp32] studied of 1 μM indicating a continual increase of the protein density along the DNA. As this protein concentration exceeds by ∼1000-fold the *K*_D_ ∼1 nM of the cooperative binding to bare ssDNA at the same force, this continued binding must correspond to a drastically different and much weaker gp32 binding mode. Using the values of the gp32–ssDNA contour length measured by our DNA stretching experiments (Figure 3C) we calculate the structural parameters of the protein-DNA helical filaments as functions of free protein concentration (Figure 9, Supplementary Table S8). These results imply that the gp32 filament on ssDNA is not a unique rigid structure, but rather a continuum of the helical structures with pitch increasing upon additional protein binding, accompanied by progressive helix unwinding, destabilization and weakening of the protein–protein contacts (Figure 10A).

This hypothesis implies two things: (i) ssDNA within its binding groove in the gp32 core must be able to move somewhat freely and optimize its position as the gp32 proteins twist relative to each other and (ii) the highly cooperative protein-protein contacts in the filament must be almost neutral with respect to the twist of one protein relative to another, i.e. gp32 proteins can twist around the filament axis with respect to one another, accommodating between ∼75° twist per protein down to ∼40° twist per protein upon ssDNA overcrowding. However, there is clearly a preferred twist angle between contiguously bound gp32, likely corresponding to the optimal protein density on ssDNA of one gp32 monomer per ∼7 nt. Moreover, there appears to be a critical lowest twist angle of the protein filament on ssDNA corresponding to ∼7 gp32 monomers per helical turn (Figure 9) at which the gp32–gp32 interactions largely vanish, giving rise to the fast (timescale of ∼50 s at 15 pN) noncooperative gp32 dissociation mode not only from the filament ends, but from the whole length of the complex (Figure 8A). This fast dissociation continues until the torsional stress of the excess proteins in the filament is relieved below its critical value. At this point the gp32–ssDNA filament becomes stable and cooperative again, leading to much slower gp32 dissociation only from its few ends.

The above assumptions about the gp32–ssDNA filament (ssDNA free motion in the complex and variability of the gp32 filament pitch on ssDNA) are non-trivial. With regard to the first point, gp32-oligonucleotide binding affinity measurements using proteolysis and DNA *T*_m_ depression methods revealed that at least two, and likely three, adjacent phosphodiester bonds are required for binding (30). This study also reported an increase in affinity of the gp32 core domain for ssDNA when the oligonucleotides (d*T_n_*) were increased in length from 5 to 8 nt suggesting that the number of interactive residues within the core can be somewhat variable and dependent on substrate length. Additionally, fluorescence measurements showed a ‘tight binding’ interaction between the gp32 core and 2–3 nt of ssDNA, and Jose et al. proposed that under monomer binding conditions the protein can fluctuate between a partially bound and fully bound state depending on the orientation of the C-terminal arm, suggesting that the gp32 core can adopt multiple conformations on ssDNA which engage different numbers of nucleotides (16,17,22,52).

Additionally, the crystal structure of the gp32 core domain in complex with a short 6-mer ssDNA lattice showed weak electron density for the DNA within the protein's binding cleft as well as some rotational and translational freedom about the phosphate backbone, suggesting that the ssDNA is fairly mobile within the gp32 binding groove (10). Other studies support this conclusion, showing that gp32 can translocate or slide freely along ssDNA either as single noncontiguous monomers or as small cooperatively-bound clusters (25,53,54). Thus, the requirement that the ssDNA is mobile and can bind gp32 in different conformations appears to be consistent with experimental evidence.

With respect to the flexibility of the gp32–gp32 interaction in cooperative binding to ssDNA and filament formation, this process primarily involves interaction of the positively-charged N-terminal domain of one protein with a (presumably) negatively-charged surface of the core domain of an adjacent DNA-bound (contiguous) protein; however, this does not exclude the possibility of additional secondary contacts between the cores of contiguously-bound gp32 (45–47). The domains are defined by the susceptibility of the full-length protein to trypsin cleavage, and the cleavage sites are presumably located at unstructured regions of the polypeptide. The N-terminus itself is largely α-helical (based on CD data and Chou-Fasman calculations) (12), but the accessibility of the NTD-core cleavage site to proteases suggests that the polypeptide chain in the vicinity of this site is likely to be quite flexible. Thus, it is conceivable that this domain is able to adopt different orientations while binding to the same surface on the adjacent gp32 core domain (3,55). This flexibility would not constrain, indeed it could accommodate, additional secondary core-core contacts. Moreover, CD spectroscopy and quasi-elastic light scattering experiments have shown a range of nucleotide-nucleotide distances along the helix axis between 0.43 and 0.56 nm (42,43). In comparison, our stretching data revealed an increase in the gp32–ssDNA contour length from 0.41 to 0.49 nm/nt as a function of protein concentration, in reasonable agreement with these previous studies. Thus, the assumption that the gp32 filament helical pitch on ssDNA can have a range of values, *i.e*. the protein twist angle is not fixed, but rather variable, is certainly plausible and supported by experiment.

As gp32 has been intensely studied over the last 50 years, it is worth considering why this behavior, namely gp32’s ability to bind ssDNA in an overextended and unstable conformation, has not been observed previously. There are several significant differences between the methodologies used here and those used previously to characterize gp32–ssDNA interactions that could account for this. First, a large majority of gp32 studies have utilized relatively short DNA substrates to measure gp32 binding/dissociation dynamics, generally ranging from a few nucleotides in length to, at most, a couple hundred nucleotides. While a considerable amount of insight can be gleaned from binding studies performed with short DNA constructs, these studies are limited to the dynamics of either singly-bound gp32 monomers or relatively small clusters thereof. In contrast, our experiments probe gp32 interactions with an ∼8 knt long ssDNA molecule, and thus reveal the large-scale, collective behavior of > 1000 proteins on a single lattice. It is entirely plausible that gp32 dynamics differ drastically between these DNA length scales due to changes in both average cluster size and the number of protein clusters present at any given time. Our measurements do, however, recapitulate gp32 behavior seen in earlier studies, *e.g*. dissociation of cooperatively-bound proteins from the ends of the stable filaments. Second, the length changes associated with the conformational transitions observed within this study are small compared to the total length of the substrate (∼10% of the total length). Thus, protein-DNA conformational measurements, such as those made with FRET, may not have the resolution to properly distinguish between the gp32 binding modes observed here. Additionally, the short DNA substrates often used in these studies likely compound this issue, making it difficult to differentiate these length changes from background. Optical tweezers systems using long ssDNA constructs, on the other hand, provide high signal-to-noise, and thus sufficient resolution to probe small-scale conformational changes (<0.005 nm/nt) that may otherwise be undetectable in other systems. Lastly, the unstable gp32 binding mode observed in this study is approximately three orders of magnitude weaker than the primary cooperative binding mode to bare ssDNA, and thus requires protein concentrations ∼1000-fold above *K*_D_ to effectively measure. The results presented here were performed with protein in great excess to the DNA, facilitating measurements of conformational changes induced by high gp32 concentrations. However, this is not necessarily true for other studies, and thus may be a reason such behavior has not been observed until now. While these conditions appear rather extreme, the concentrations needed to observe these binding effects fall within the range of gp32 concentrations found *in vivo* and are therefore consistent with behavior expected within the cell. Moreover, very high protein concentrations may not be entirely necessary to trigger gp32’s unstable binding mode. Our results suggest that transition into this binding state is driven by increases in gp32 density on the DNA. As discussed below, ssDNA length changes, such as those that occur during movement of the replication fork, may also increase protein density along the strand, and in turn create conditions that mimic those produced by high concentrations of free protein as seen in this study.

### Functional role of unstable gp32 binding state during DNA replication

gp32’s high affinity binding and sequence non-specificity enable efficient coating of ssDNA regions transiently formed during DNA replication, offering protection from enzymatic degradation. Moreover, its ability to effectively discriminate against duplex DNA stimulates replisome processivity by melting out adventitious secondary structure (56). Conversely, gp32 must also allow the DNA to be accessed by T4 polymerase as well as other constituents of the T4 recombination, replication, and repair machinery. However, high protein cooperativity prevents gp32 from being easily displaced from ssDNA substrates, raising the question as to how these tightly-bound filaments undergo rapid reorganization, e.g. protein dissociation, required for genomic maintenance processes.

Given that the gp32 concentration in T4-infected *E. coli* is autogenously regulated at ∼2–3 μM (6–8), a level sufficient to completely saturate all available ssDNA, the mechanism of sliding along bare DNA may not contribute significantly to the rapid reorganization required to keep up with the moving polymerase. It is unlikely that very large protein clusters can translocate fast enough (if at all) to maintain the observed rate of DNA synthesis, and the subsequent primer at the end of each Okazaki fragment would provide a barrier to ssDNA-specific binding. Salt jump measurements of gp32 dissociation by Peterman et al. (57) suggested that long protein clusters can peel off of the DNA in an ‘all or nothing’ manner similar to the highly cooperative helix-coil transitions of polynucleotides, potentially representing a rapid and efficient mechanism for displacement of gp32 during complementary strand synthesis. However, the apparent dissociation rate constant measured in that study was still well below the estimated *in vivo* fork rate of 400–700 bp/s (23). Additionally, it is unclear if perturbations similar to those experienced during salt jump measurements actually occur near the replication fork during DNA synthesis, and thus if this dissociation scheme is plausible.

Taken together, the results described within this study provide a possible alternative mechanism for rapid removal and recycling of gp32 during complementary strand polymerization (Figure 9B). Our measurements suggest that transiently formed ssDNA regions, such as Okazaki fragments, are immediately saturated by free gp32, forming tightly-bound, filamented complexes. Previous work on gp32–ssDNA interactions reported an approximate association rate of 15–20 s^−1^ (24,25), in reasonable agreement with the rapid binding and compaction observed within this study. Following coating, the ssDNA-bound gp32 are presumably in a highly stable conformation and, thus, not easily removed from the substrate. Indeed, ours and previous measurements (24) show that the unperturbed lifetimes of singly contiguously bound proteins are far too long (*i.e*. dissociation from cluster ends is too slow) for efficient turnover during the replication process. Nonetheless, it is possible that gp32 is actively displaced by the moving replication complex in a sequential manner from the ends of the protein cluster. Such a mechanism is supported by studies demonstrating species-specific interprotein interactions between gp32 and T4 polymerase that strongly stimulate *in vitro* DNA synthesis rates (27,28). However, considering our evidence that gp32 can rapidly dissociate across the entire substrate, it is also plausible that active displacement of gp32 by T4 polymerase combined with gp32’s ability to slide along the ssDNA increases the protein density on the substrate as the Okazaki fragment shortens. This could, in turn, drive the complex into an oversaturated state, presumably mimicking the overextended state that we observed under conditions of high protein concentration (Figure 10). Our measurements showed that this conformation was significantly less stable, exhibiting rapid dissociation at least an order of magnitude faster than subsequent unbinding from the ends of the protein clusters. Notably, this dissociation phase was exponential, indicating that gp32 overcrowding on the template strand can be relieved by dissociation of any gp32 across the entire ssDNA segment, facilitating faster displacement of gp32, and thereby clearing the way for complementary strand synthesis without the need for sliding of the whole cooperative gp32 filament or its de-polymerization from the end. Moreover, as the ssDNA template becomes shorter this process may continue, regulating the gp32 density and allowing T4 polymerase to proceed while ensuring maximal coverage of the ssDNA at all times.

Within T4-infected *E. coli*, second strand DNA synthesis by the replication complex occurs at a rate of ∼500 nt/s (23). Moreover, the length of the Okazaki fragments of ssDNA templates is 1000–2000 nt, i.e. significantly longer than the typical Okazaki fragments in eukaryotes that are only 100–300 nt long (58). Our proposed mechanism of facilitated gp32 dissociation via its overcrowding in front of the moving replication fork becomes more efficient on longer ssDNA templates. This is because the dissociation from such destabilized filaments becomes noncooperative and the total number of proteins dissociating per unit time is proportional to the filament length. Assuming prompt re-equilibration of the ssDNA winding on the remaining relaxed filament that keeps ssDNA gp32-engaged and protected from nucleases at all times, this mechanism can lead to rapid template clearing that is faster on the longer template. This is in contrast to the two alternative models: (i) of the whole gp32 filament moving in front of the polymerase that must be very slow, and (ii) gp32 dissociation from the few ends of highly cooperative filaments, with the typical off time ∼500 s, which is independent of the ssDNA template length. Our fitted non-cooperative gp32 monomer dissociation rate from the overcrowded filament at 1 μM gp32 in solution at 15 pN is ∼0.02 s^−1^, i.e. the protein dissociates over ∼50 s. Assuming that each gp32 binds ∼7 nt, about ∼70 gp32 proteins per second must dissociate from the complex to clear the way for the polymerase synthesizing the complementary strand at a rate of ∼500 nt/s. As a ∼2000-long Okazaki fragment of ssDNA template binds ∼300 gp32 protein at saturation with each of these proteins dissociating from the overcrowded template with rate *k*, clearing of the 70 proteins per second from such a long template can be achieved when the rate of individual protein noncooperative dissociation reaches *k* = 70 s^−1^/300 ∼ 0.2 s^−1^. While this rate is still about 10-fold higher than what we have measured for the dissociation from the overcrowded filament under our *in vitro* conditions (1 μM gp32 and 15 pN force), it is entirely possible that this rate is achieved *in vivo* under physiological conditions. Indeed, typical gp32 concentrations in T4-infected *E. coli* are maintained at ∼2–3 μM (6–8), which may lead to even stronger gp32–ssDNA filament oversaturation and faster protein dissociation. It is also plausible that in the absence of tension on the filament its oversaturation with gp32 is yet stronger and the dissociation faster than at 15 pN tension used in our experiments. Moreover, movement of the replication fork coupled with interactions between gp32 and T4 polymerase may facilitate further overcrowding along the template strand, thereby stimulating faster removal of gp32. In conclusion, our proposed mechanism, in which polymerase-induced overcrowding on long ssDNA facilitates prompt gp32 dissociation, could potentially be the primary mode for ssDNA clearing during DNA replication. At the same time, the proposed gp32 dissociation mechanism never leaves ssDNA unprotected, as this dissociation never removes all gp32 from the template, but only relieves it of its overcrowding, relaxing the complex to a completely saturated enzyme-protected state.

The gp32 binding dynamics measured in this study share many similarities with those of the *E. coli* ssDNA binding protein (*Ec*SSB). In particular, both proteins exhibit multiphasic ssDNA extension profiles regulated by the protein density along the DNA, with additional binding into the saturated complex resulting in protein overcrowding, facilitating transition into a less compact and less stable state that dissociates rapidly upon removal of free protein (33). Strikingly, however, the structures of these two SSBs are markedly different. Whereas gp32 is primarily monomeric in solution and oligomerizes on the ssDNA substrate, *Ec*SSB forms tetramers in solution and does not form higher order oligomers. As a result, the various binding modes of *Ec*SSB, which arise from the ssDNA directly binding to a different number of tetramer subunits, can be observed for even single proteins (59). In contrast, gp32 forms highly cooperative, continuous filaments that helically wind ssDNA. gp32’s ability to wind ssDNA in multiple conformations appears to be purely a consequence of collective dynamics across the entire filament (*i.e*. the modulation of cooperative interprotein interactions that alter the twist angle between adjacent gp32 molecules, leading to the variation in helical pitch of the protein-DNA filament) rather than the intrinsic behavior of single, isolated proteins. Thus, while the underlying mechanisms of *Ec*SSB and gp32 binding differ significantly from one another, both proteins give rise to comparable collective behavior on long ssDNA templates. Indeed, Naufer *et al.* proposed a similar model for the efficient removal and recycling of *Ec*SSB during DNA replication—polymerase-driven overcrowding along the template strand facilitates transition to a less wrapped and less stable protein state that dissociates rapidly across the entire substrate, allowing for fast displacement of the strongly wrapped tetramers (33). Our showing that different types of structural interactions, from highly cooperative monomers to strongly wrapping tetramers, can exhibit comparable collective dynamics suggests that our proposed model may be generalizable to other SSBs.

## Supplementary Material

gkad595_Supplemental_File

## Data Availability

The experimental data sets are either included in the main text, supplementary materials, or are available from the authors upon request.
