# Correction to ‘Dynamic structure of T4 gene 32 protein filaments facilitates rapid noncooperative protein dissociation’

**DOI:** 10.1093/nar/gkad780

**Published:** 2023-09-20

*Nucleic Acids Research*, Volume 51, Issue 16, 8 September 2023, Pages 8587–8605, https://doi.org/10.1093/nar/gkad595

In the originally published version of this manuscript, there was a small error in the second column of page 8599: …‘pitch increases according to (3)…’ should say ‘according to Eq. (4)’.

This error has been corrected online.

