# Supplementary material for: Dynamic structure of T4 gene 32 protein filaments facilitates rapid noncooperative protein dissociation
Source: Nucleic Acids Res. 2023 Jul 14;51(16):8587–605. doi: 10.1093/nar/gkad595 (PMC10484735; doi:10.1093/nar/gkad595)
Supplement: gkad595_Supplemental_File [file gkad595_supplemental_file.pdf]

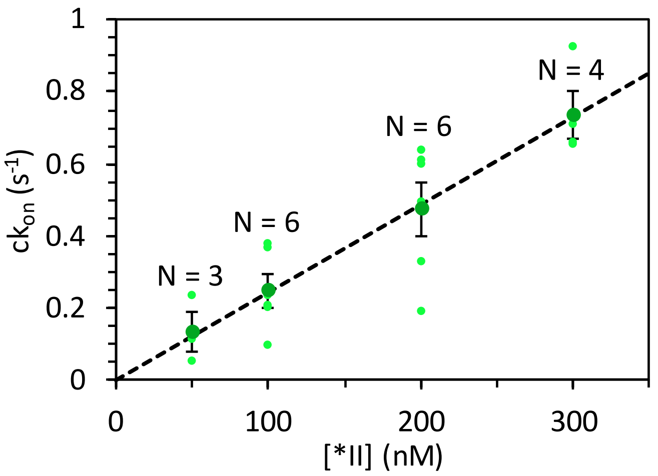

Figure S1: *II binding rate. Average rate of protein binding (green, replotted from Fig. 2B) with respect to concentration. Individual data points (light green) are shown with the number of replicates (N) for each concentration.

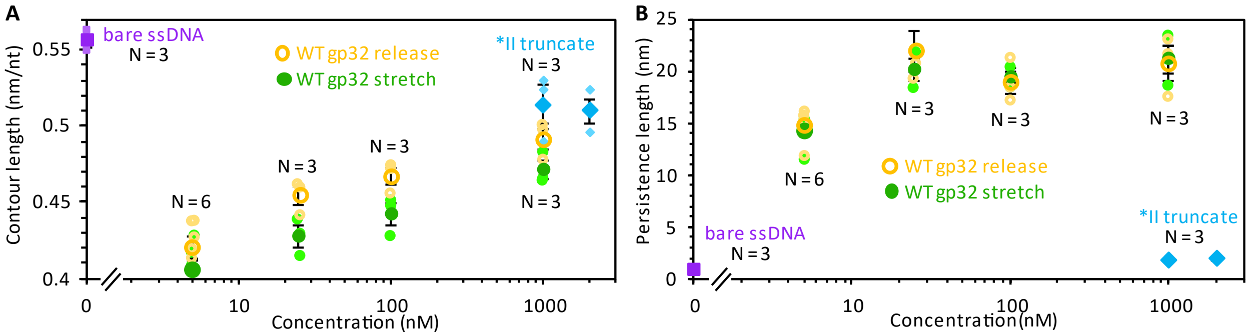

Figure S2: Contour and persistence lengths of gp32-ssDNA complexes. (A) Average contour length of bare ssDNA (purple square) and ssDNA saturated with *II (blue diamonds) and WT gp32 during stretch (green filled circles) and release (yellow empty circles) with respect to protein concentration (replotted from Fig. 3C). Individual data points are shown (light colors) with the number of replicates (N) for each condition. (B) Average persistence length of bare ssDNA (purple square) and ssDNA bound with *II (blue diamonds) and WT gp32 during stretch (green filled circles) and release (yellow empty circles) with respect to protein concentration (replotted from Fig. 3D). Individual data points are shown (light colors) with the number of replicates (N) for each condition.

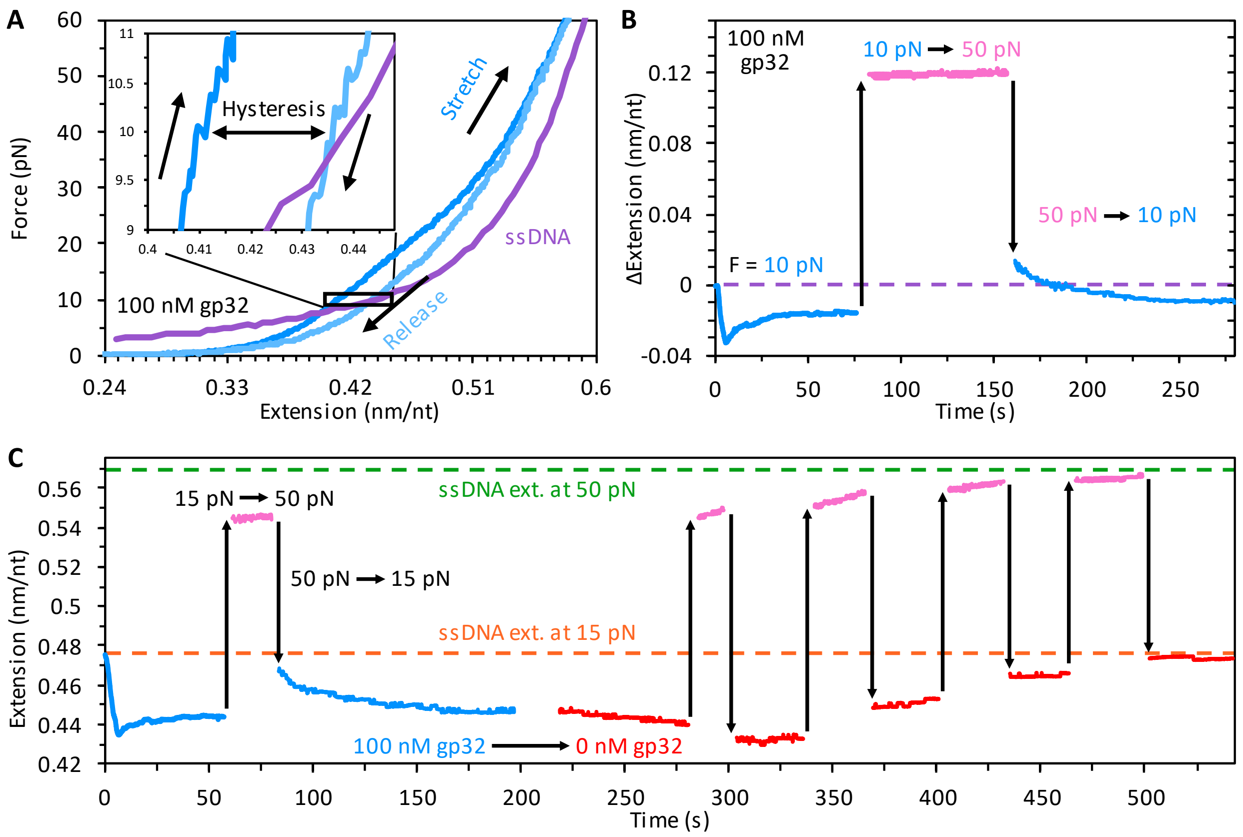

Figure S3: Probing gp32-ssDNA hysteresis. (A) The ssDNA was slowly stretched (blue) in the presence of 100 nM gp32. Upon release (light blue), the protein-DNA complex exhibits hysteresis (inset shows hysteresis at 10 pN). (B) The ssDNA was initially incubated with 100 nM gp32 at a fixed force of 10 pN (blue, extension changes are shown relative to bare ssDNA at 10 pN). Following equilibration, the tension on the DNA was increased to 50 pN for ~100 seconds (pink). The tension was then lowered back to 10 pN (blue) where the complex exhibited a phase of exponential compaction, re-equilibrating to an extension slightly longer than that seen prior to the increase in force (purple dashed line serves as a reference for bare ssDNA at 10 pN). (C) The ssDNA was initially incubated with 100 nM gp32 at a fixed force of 15 pN (blue). Following equilibration, the tension on the DNA was increased to 50 pN for ~20 seconds (pink). The tension was then lowered back to 15 pN (blue) where the complex experienced a phase of exponential compaction. Free protein was replaced with protein-free buffer leading to further (linear) DNA compaction (red) as gp32 dissociated. The tension on the substrate was subsequently cycled between 15 and 50 pN repeatedly. During cycling the ssDNA first becomes more compact at 15 pN (red) before extending toward its protein-free conformation. In contrast, the overall extension change at 50 pN (pink) is single-phased. The extensions of bare ssDNA at 15 pN (dashed orange line) and 50 pN (dashed green line) are indicated for reference.

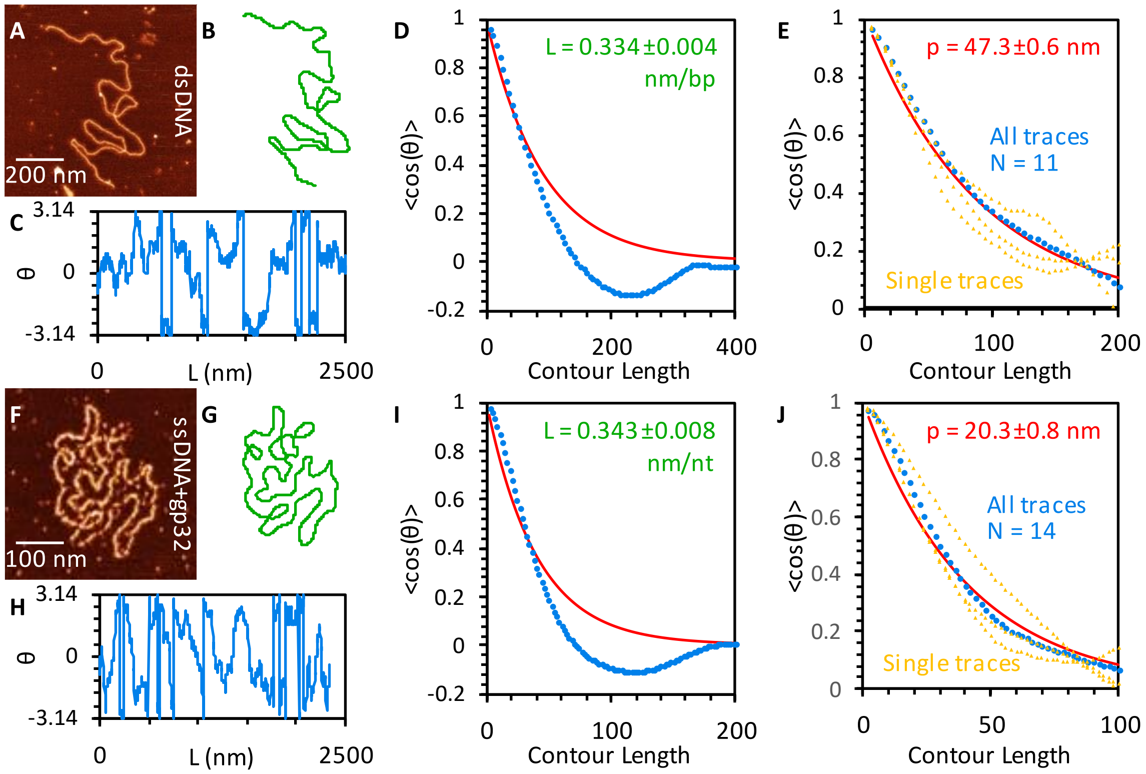

Figure S4: Measuring contour and persistence length. (A) AFM image of 7249 bp linear dsDNA shows WLC-like behavior with two self-intersections. (B) A trace of the molecule is produced consisting of points spaced 2 nm apart following the DNA backbone. (C) The instantaneous angle of the dsDNA is plotted along its length. (D) The average value of cos(θ) is calculated for every two points in the trace separated by a distance L, then averaged again over all traces of individual dsDNA molecules (blue data points). While the full-length trace returns an accurate value for dsDNA contour length, the angle measurements show a negative correlation near 200 nm, caused by loops formed by the dsDNA, preventing a good fit by an idealized WLC model (red line). (E) The cos(θ) values are recalculated along its length using the trace from the free ends up to the first dsDNA self-intersection. Averaging cos(θ) values over only a single DNA construct (yellow, 3 molecules plotted) yields noisy results, especially at longer distances, due to a limited number of DNA persistence lengths in each trace, but averaging all molecules together produces a large enough data set to accurately measure this trend. Omitting the looped regions of dsDNA returns an exponential decay in angle correlation, allowing fitting by the WLC model that returns a persistence length value consistent with previous measurement. (F-J) Analysis is repeated for ssDNA incubated with 100 nM gp32. Tracing the entirety of each molecule returns the measurable contour length of the gp32-ssDNA complex (I), while tracing only the longest segment of each molecule without self-intersection returns the effective persistence length of the complex (J).

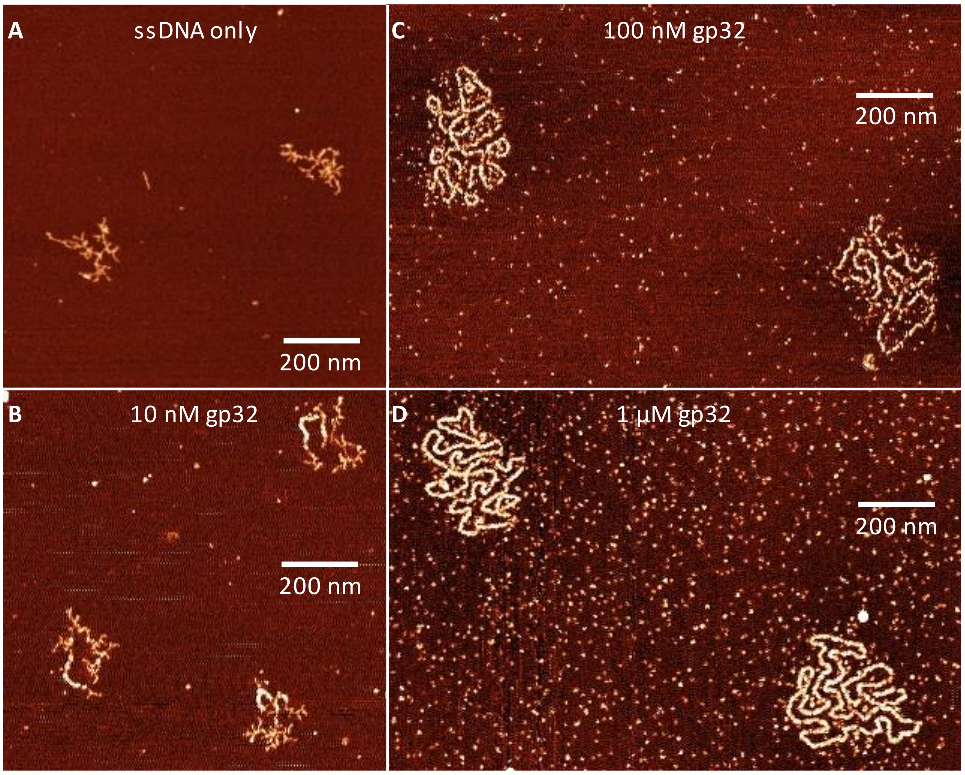

Figure S5: ssDNA constructs are imaged by AFM in the absence (A) and presence (B-D) of gp32. All images are presented at the same lateral and height scale (maximum intensity height is 2 nm) for comparison. 10 nM gp32 binds the ssDNA, as evidenced by increased pixel brightness and volume (Fig. 4C) but does not sufficiently linearize the DNA to allow accurate tracing.

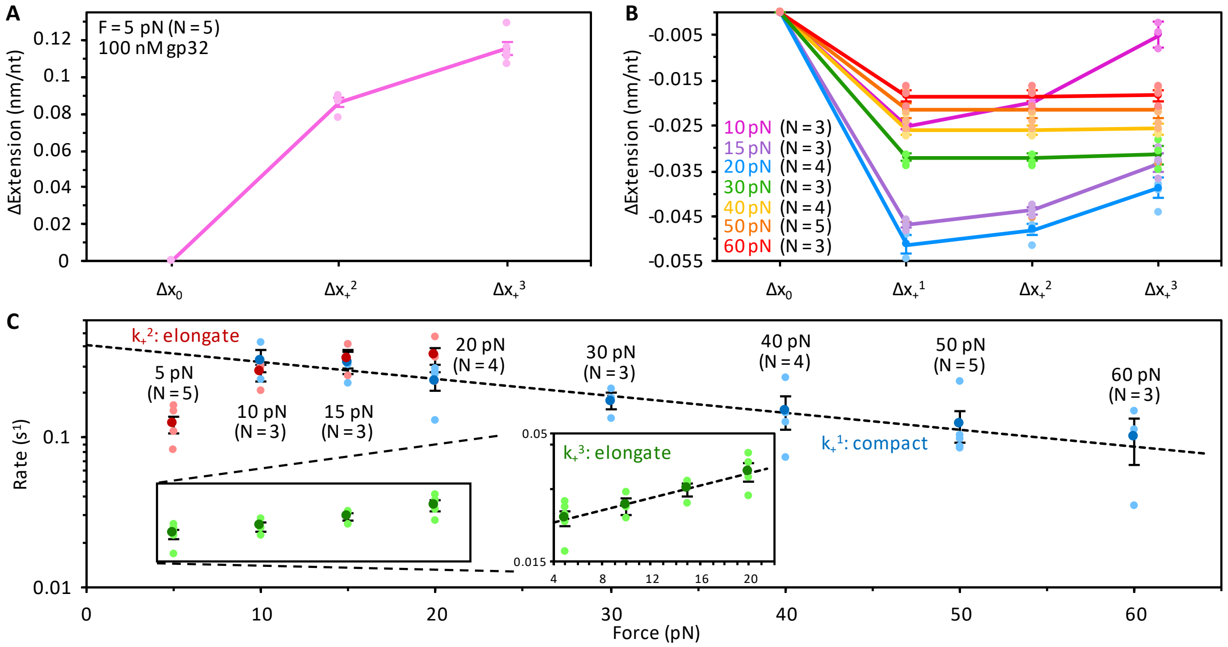

Figure S6: Force dependence of gp32 binding. (A) Average extension changes of ssDNA in the presence of 100 nM gp32 at 5 pN (pink, replotted from Fig. 5A). Individual data points (light pink) are shown for five replicate measurements. (B) Average ssDNA extension changes in the presence of 100 nM gp32 as functions of DNA tension (replotted from Fig. 5B). Individual data points (light colors) are shown with the number of replicates (N) for each force. (C) Average ssDNA compaction and elongation rates as functions of DNA tension (replotted from Fig. 5C). Individual data points (light colors) are shown with the number of replicates (N) for each force.

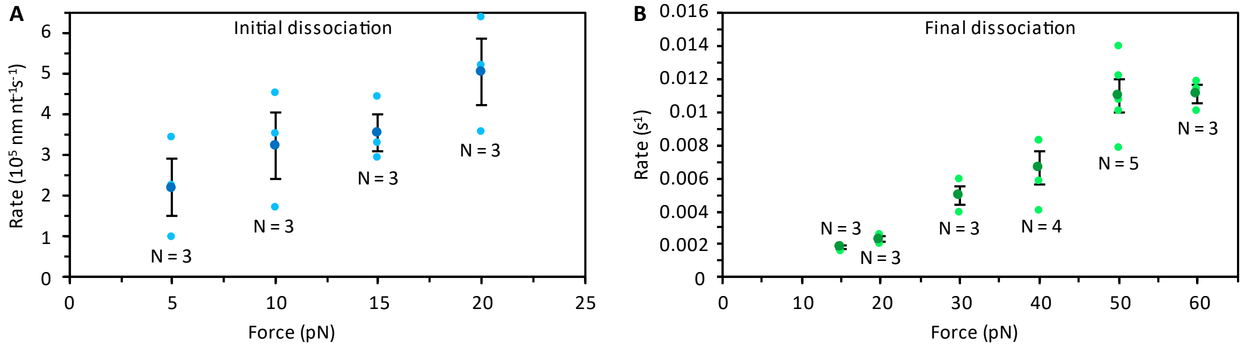

Figure S7: Force dependence of gp32 dissociation. (A) Average rate of (linear) ssDNA recompaction during initial dissociation of gp32 (blue, replotted from Fig. 6C) as a function of DNA tension. Individual data points (light blue) are shown with the number of replicates (N) for each force. (B) Average rate of ssDNA elongation during the final gp32 dissociation phase (green, replotted from Fig. 6D) as a function of DNA tension. Individual data points (light green) are shown with the number of replicates (N) for each force.

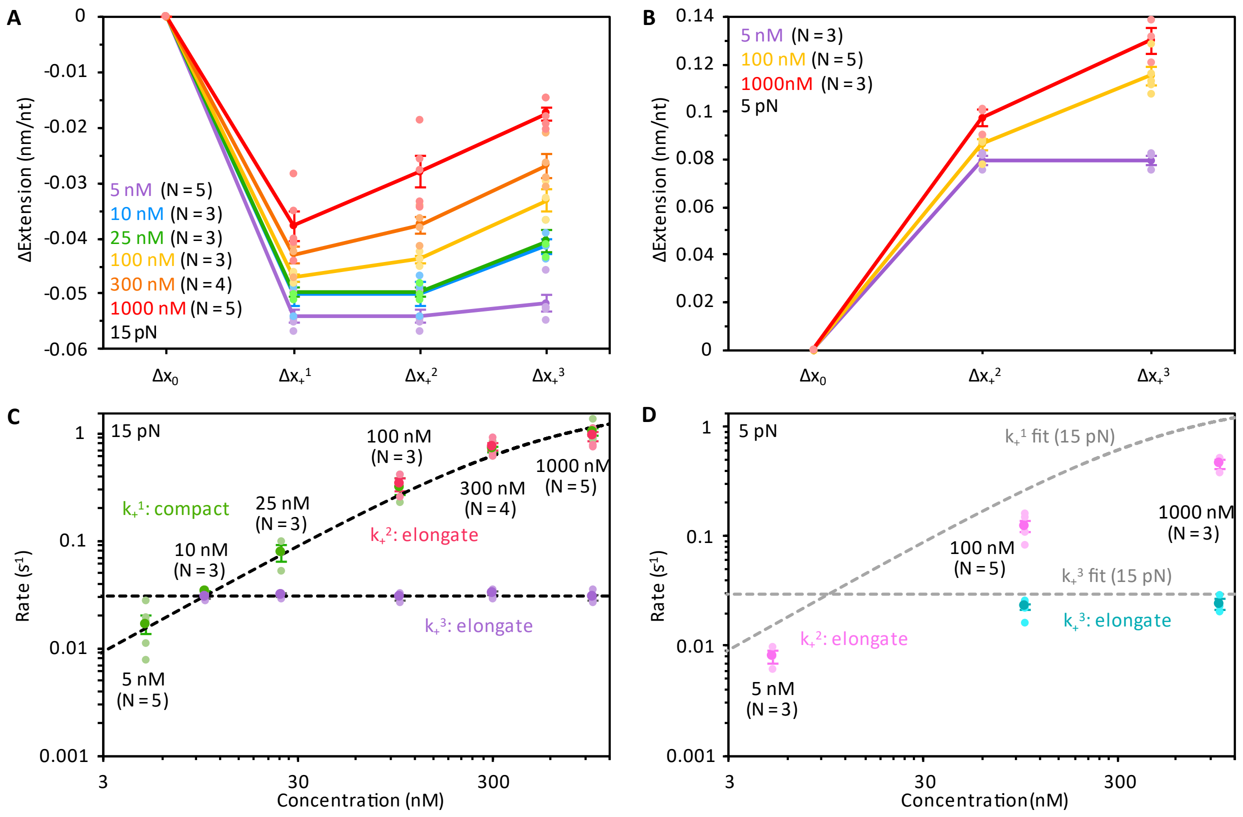

Figure S8: Concentration dependence of gp32 binding. (A) Average ssDNA extension changes at 15 pN as functions of protein concentration (replotted from Fig. 7A). Individual data points (light colors) are shown with the number of replicates (N) for each concentration. (B) Average ssDNA extension changes at 5 pN as functions of protein concentration (replotted from Fig. 7B). Individual data points (light colors) are shown with the number of replicates (N) for each concentration. (C) Average ssDNA compaction and elongation rates as functions of protein concentration at 15 pN (replotted from Fig. 7C). Individual data points (light colors) are shown with the number of replicates (N) for each concentration. (D) Average ssDNA elongation rates as functions of protein concentration at 5 pN (replotted from Fig. 7D). Individual data points (light colors) are shown with the number of replicates (N) for each concentration.

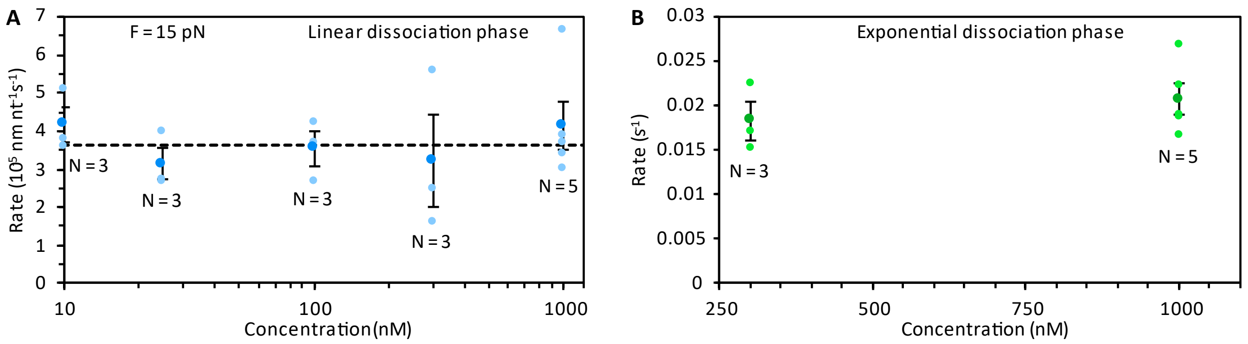

Figure S9: Concentration dependence of gp32 dissociation. (A) Average rate of linear recompaction phase (blue, replotted from Fig. 8B) during initial gp32 dissociation as a function of protein incubation concentration. Individual data points (light blue) are shown with the number of replicates (N) for each incubation concentration. (B) Average rate of exponential recompaction phase (green, replotted from Fig. 8C) during initial gp32 dissociation as a function of protein incubation concentration. Individual data points (light green) are shown with the number of replicates (N) for each incubation concentration.

F = 15 pN	Δx_{*II} (nm/nt)	k_{on} ($nM^{-1}s^{-1}$)	k_{off} (s^{-1})	K_D (nM)
	-0.013±0.001	0.0024±0.0002	0.11±0.01	46±6

Table S1: *II binding/dissociation measurements. Average extension change and rates associated with binding and dissociation of the *II truncate at 15 pN (see Fig. 2). The on-rate was calculated by fitting ck_{on} ($ck_{on} = k_{obs} - k_{off}$) as a function of concentration to a straight line. Additionally, $K_D = k_{off}/k_{on}$.

	ssDNA	*II	gp32 (stretch)				gp32 (release)			
c (nM)	0	2000	5	25	100	1000	5	25	100	1000
L (nm/nt)	0.556 ±0.004	0.510 ±0.008	0.405 ±0.007	0.428 ±0.007	0.442 ±0.007	0.471 ±0.006	0.420 ±0.008	0.454 ±0.006	0.467 ±0.006	0.492 ±0.007
p (nm)	0.8±0.1	1.9±0.1	14.2±0.6	20.1±1.0	19.4±0.6	21.1±1.4	14.8±0.6	21.9±2.0	19.0±1.2	20.7±1.7

Table S2: Comparison of contour and persistence lengths. Average contour and persistence length measurements of bare ssDNA and ssDNA saturated with *II and WT gp32 (see Fig. 3). The ssDNA was slowly (~10 nm/s) stretched and released in the presence of various concentrations of *II and WT gp32. The resulting *II force-extension curve was fit with the freely jointed chain up to 10 pN to compute the contour and persistence lengths. The force-extension curves of WT gp32 were fit with the worm-like chain up to 5 pN to calculate the contour and persistence lengths during stretch and release.

Force (pN)	Δx_+^1 (nm/nt)	k_+^1 (s^{-1})	Δx_+^2 (nm/nt)	k_+^2 (s^{-1})	Δx_+^3 (nm/nt)	k_+^3 (s^{-1})
5	N/A	N/A	0.086±0.002	0.12±0.01	0.115±0.004	0.023±0.002
10	-0.025±0.001	0.33±0.06	-0.020±0.001	0.27±0.03	-0.005±0.003	0.026±0.002
15	-0.047±0.001	0.32±0.06	-0.044±0.001	0.33±0.05	-0.033±0.002	0.030±0.002
20	-0.051±0.002	0.24±0.04	-0.048±0.001	0.35±0.05	-0.039±0.002	0.031±0.003
30	-0.032±0.001	0.17±0.02	-0.032±0.001	N/A	-0.031±0.002	N/A
40	-0.026±0.001	0.15±0.04	-0.026±0.001	N/A	-0.026±0.001	N/A
50	-0.022±0.002	0.12±0.03	-0.022±0.002	N/A	-0.021±0.002	N/A
60	-0.018±0.001	0.10±0.03	-0.018±0.001	N/A	-0.018±0.001	N/A

Table S3: Force-dependent binding measurements. Average extension changes and rates associated with each gp32 binding phase as functions of ssDNA tension (see Fig. 5). All data were taken with 100 nM gp32. No compaction phase (Δx_+^1) was observed at 5 pN tension. Additionally, multiphasic extension changes were only observed at forces ≤ 20 pN, above which the ssDNA compaction was single-phased.

Force (pN)	k_{-}^{1} (10^{-5} nm $nt^{-1}s^{-1}$)	k_{-}^{2} (10^{-3} s^{-1})
5	2.2±0.7	N/A
10	3.2±0.8	N/A
15	3.5±0.5	1.8±0.1
20	5.0±0.8	2.3±0.1
30	N/A	5.0±0.6
40	N/A	6.6±1.0
50	N/A	11.0±1.0
60	N/A	11.1±0.6

Table S4: Force-dependent dissociation measurements. Average rates associated with each gp32 dissociation phase as functions of ssDNA tension (see Fig. 6). All data were taken subsequent to incubation with 100 nM gp32. Initial recompaction (k_{-}^{1}) was only observed at forces ≤ 20 pN. At forces ≤ 10 pN, the final dissociation phase was too slow to accurately measure within the timescale of the experiment.

Concentration (nM)	Δx_+^1 (nm/nt)	k_+^1 (s^{-1})	Δx_+^2 (nm/nt)	k_+^2 (s^{-1})	Δx_+^3 (nm/nt)	k_+^3 (s^{-1})
5	-0.054±0.001	0.017±0.003	-0.054±0.001	N/A	-0.052±0.002	N/A
10	-0.050±0.002	0.034±0.001	-0.050±0.002	N/A	-0.041±0.001	0.030±0.001
25	-0.050±0.001	0.076±0.013	-0.050±0.001	N/A	-0.041±0.002	0.031±0.001
100	-0.047±0.001	0.317±0.055	-0.044±0.001	0.33±0.05	-0.033±0.002	0.030±0.001
300	-0.043±0.001	0.702±0.049	-0.038±0.002	0.75±0.07	-0.027±0.002	0.032±0.001
1000	-0.038±0.003	1.040±0.086	-0.028±0.003	0.93±0.07	-0.017±0.001	0.030±0.001

Table S5: Concentration-dependent binding measurements at 15 pN. Average extension changes and rates associated with each gp32 binding phase as functions of protein concentration (see Fig. 7). All data were taken at 15 pN tension. gp32 binding and compacting rates were calculated by fitting the concentration-dependent compaction rate (k_+^1) to a two-rate equation: $k_+^1 = [1/(ck_b) + 1/k_c]^{-1}$ where c is the protein concentration, k_b is the gp32 binding rate, and k_c is the compacting rate. Rapid partial elongation (k_+^2) was only observed at concentrations ≥ 100 nM. At 5 nM, the ssDNA compaction was single-phased.

Concentration (nM)	Δx_+^2 (nm/nt)	k_+^2 (s^{-1})	Δx_+^3 (nm/nt)	k_+^3 (s^{-1})
5	0.080±0.002	0.008±0.001	0.080±0.002	N/A
100	0.086±0.002	0.122±0.015	0.115±0.004	0.023±0.002
1000	0.097±0.004	0.458±0.043	0.130±0.005	0.024±0.002

Table S6: Concentration-dependent binding measurements at 5 pN. Average extension changes and rates associated with each gp32 binding phase as functions of protein concentration (see Fig. 7). All data were taken at 5 pN tension. At 5 nM, the ssDNA elongation was single-phased.

Concentration (nM)	k_{-1} (10^{-5} nm $nt^{-1}s^{-1}$)	k_{-1}^* (s^{-1})
10	4.2±0.5	N/A
25	3.1±0.4	N/A
100	3.5±0.5	N/A
300	3.2±1.2	0.018±0.002
1000	4.1±0.7	0.021±0.002

Table S7: Concentration-dependent dissociation measurements. Average rates associated with the initial gp32 dissociation phase (recompaction) as functions of protein incubation concentration (see Fig. 8). All data were taken at 15 pN tension. The initial rapid dissociation phase (k_{-1}^*) was only observed subsequent to incubating the ssDNA with protein concentrations ≥ 300 nM.

Concentration (nM)	L/L′	bss (nt/protein)	α (°/protein)	ρ (nm/turn)	N (protein/turn)	Density (protein/nt)
5	1.37±0.03	7	73±4	13.9±0.6	4.9±0.3	0.143
25	1.30±0.02	6.6±0.2	65±3	15.8±0.7	5.6±0.3	0.151±0.004
100	1.26±0.02	6.4±0.2	60±3	17.1±0.8	6.0±0.3	0.156±0.004
1000	1.18±0.02	6.0±0.1	49±3	20.8±1.0	7.3±0.4	0.166±0.004
*1000	1.13±0.02	5.7±0.1	41±2	24.8±1.2	8.8±0.5	0.174±0.004

Table S8: Concentration-dependent parameters of gp32-ssDNA helix. Average structural parameters of gp32-ssDNA helix as functions of free protein concentration (see Fig. 9). * denotes helical parameters associated with the longest observed gp32-DNA contour length measured at 1 μM [gp32] during release of the complex subsequent to stretching (Fig. 3C, open red circle).
